# Factors Influencing Diagnostic Adherence Among High-Risk Populations for Chronic Obstructive Pulmonary Disease in Eastern Rural China: An Analysis Based on the Health Belief Model and Implications for Primary Care Interventions

**DOI:** 10.3390/healthcare14193251

**Published:** 2026-10-01

**Authors:** Zhongli Wang, Gaopei Zhu, Jin Xie

**Affiliations:** 1 Institute of Non-Communicable Chronic Disease Control and Prevention, Beijing Center for Disease Prevention and Control , Beijing 100013, China; 2 Shandong Mental Health Center, Jinan 250000, China

**Keywords:** Chronic Obstructive Pulmonary Disease (COPD), diagnostic adherence, Health Belief Model (HBM), social support, economic toxicity, rural population, health literacy, primary care intervention, eastern China

## Abstract

**Highlights:**

**What are the main findings?**

- Health literacy, health beliefs, and COPD knowledge are positively associated with two-dimensional diagnostic adherence, while economic toxicity and living alone are core barriers among rural high-risk populations for COPD in eastern China.
- Health beliefs statistically mediate 31.4% of the association between COPD knowledge and cognitive adherence and 36.9% of the association between economic toxicity and cognitive adherence (cognitive outcome only; behavioral indirect associations are reported on the log-odds scale).

**What are the implications of the main findings?**

- Rural primary care interventions for early COPD diagnosis should simultaneously address cognitive barriers (literacy education, belief strengthening) and behavioral barriers (financial relief, improved accessibility); these are proposed as future strategies requiring prospective evaluation.
- Targeted strategies that focus on instrumental social support and indirect cost reduction are needed, especially for vulnerable subgroups such as men living alone.

**Abstract:**

Background/Objectives: Chronic Obstructive Pulmonary Disease (COPD) is a progressive respiratory disorder imposing massive global mortality and socioeconomic burden, with disproportionately high prevalence and suboptimal diagnostic rates in rural China. This study aimed to identify factors associated with two-dimensional diagnostic adherence—operationalized as a cognitive/psychological dimension (knowledge of diagnostic guidelines, perceived value of early diagnosis, and psychological readiness) and a behavioral dimension (completion of standard diagnostic procedures)—among COPD high-risk populations based on the Health Belief Model (HBM), and to explore the statistical mediating roles of health beliefs and social support. Economic toxicity refers to the financial burden of healthcare, including direct costs (copayments, medications) and indirect costs (transport, lost wages). Methods: A cross-sectional survey was conducted among 248 COPD high-risk individuals recruited via stratified random sampling in Pingdu District, Shandong Province, eastern China. A two-stage screening procedure was employed: first-stage screening at township health centers using portable spirometry, followed by referral for standard diagnostic evaluation at secondary or higher-level hospitals. Validated scales were applied for data collection, including the European Health Literacy Survey Questionnaire-Short Form (HLS-EU-Q16). Analyses included descriptive statistics, reliability/validity testing, Pearson and point-biserial correlations, multiple linear regression (cognitive outcome), multivariable logistic regression (behavioral outcome), and Hayes’ PROCESS macro with 5000 bootstrap samples. Benjamini–Hochberg false discovery rate correction was applied to multiple comparisons. Results: The mean cognitive dimension score was 4.935 ± 2.512, and the behavioral diagnostic completion rate was 42.3%. Independent predictors of cognitive adherence included health literacy (β = 0.286), health beliefs (β = 0.252), COPD knowledge (β = 0.239), living alone (β = −0.157), and economic toxicity (β = −0.108). Predictors of behavioral adherence identified via logistic regression were health literacy (OR = 1.28, 95%CI: 1.15–1.42), social support (OR = 1.12, 95%CI: 1.04–1.21), economic toxicity (OR = 0.82, 95%CI: 0.72–0.94), and medical accessibility (OR = 1.85, 95%CI: 1.12–3.05). Health beliefs statistically mediated 31.4% of the knowledge → cognitive adherence association and 36.9% of the economic toxicity → cognitive adherence association (cognitive outcome; behavioral indirect associations are reported on the log-odds scale with bootstrap confidence intervals). GAD-7 anxiety score was weakly correlated with adherence but not an independent predictor. Conclusions: Health literacy, health beliefs, and COPD knowledge are associated with higher COPD diagnostic adherence in rural high-risk populations in eastern China, while economic toxicity and living alone are associated with lower adherence. Health beliefs mediate the statistical associations between knowledge and economic status and cognitive adherence. Given the cross-sectional design, these findings should be interpreted as associations rather than causal effects. Dual-target interventions covering both cognitive and behavioral barriers are proposed as future strategies to optimize early COPD diagnosis in rural primary care.

## 1. Introduction

Chronic Obstructive Pulmonary Disease (COPD) is a progressive, heterogeneous respiratory disorder characterized by persistent airflow limitation, with global health impacts that extend beyond mortality to encompass reduced quality of life and substantial socioeconomic burden [1]. According to the Global Burden of Disease Study 2023, COPD accounts for 3.2 million annual deaths worldwide—representing 5.6% of all-cause mortality—and 2.1 billion Disability-Adjusted Life Years (DALYs), with low- and middle-income countries (LMICs) contributing 68% of these DALYs [2]. In China, the National COPD Prevalence Survey (2020) reported a prevalence of 13.7% among adults aged ≥ 40 years—surpassing the global average of 11.7%—and this burden is disproportionately concentrated in rural areas [3]. Rural Chinese populations face unique exposure to risk factors: 42% of rural households rely on biomass fuel for cooking/heating, 28% of rural men are current smokers, and occupational exposure to dust/pollutants is widespread [4].

Early diagnosis via spirometry—the gold standard for COPD diagnosis —is critical for improving patient outcomes. Meta-analyses show that early interventions (smoking cessation counseling, inhaled bronchodilators, pulmonary rehabilitation) reduce acute exacerbation risk by 30%, improve 5-year survival by 18%, and reduce annual healthcare costs by 24% [5]. However, diagnostic adherence—defined by the World Health Organization (WHO) as “the extent to which a patient’s behavior aligns with clinical recommendations”—remains suboptimal in rural China. Analysis of the national ‘Happy Breathing’ Program care cascade found that among individuals identified with COPD, only 41.0% had undergone spirometry testing and 17.6% had received a formal diagnosis, with rural residence and low regional per capita GDP associated with larger gaps at each cascade stage [6]. This diagnostic gap delays disease management, accelerates lung function decline, and increases the risk of severe exacerbations requiring hospitalization.

### 1.1. Conceptualization of Diagnostic Adherence

Diagnostic adherence for COPD high-risk populations occupies a critical niche in the chronic disease care cascade, bridging the “screening identification” and “treatment initiation” stages [7]. Traditional research on COPD diagnostic behavior has focused exclusively on behavioral outcomes—such as spirometry completion rate or healthcare-seeking frequency—and has failed to account for the cognitive and psychological antecedents that drive behavioral decisions. Systematic reviews of COPD adherence research have noted a predominant focus on behavioral outcomes such as inhaler adherence [8], with cognitive and psychological antecedents receiving less attention, and no prior study has explicitly operationalized diagnostic adherence as a dual-dimensional construct [9,10]. This oversight limits understanding of why rural populations avoid diagnostic procedures: for example, a patient may be aware of spirometry recommendations but face financial constraints, transport difficulties, or distrust in medical institutions, leading to non-adherence [11,12].

To address this limitation, we conceptualized diagnostic adherence as two interrelated dimensions, drawing on the Integrative Model of Behavioral Prediction [13] and prior chronic disease adherence frameworks:

- Cognitive/psychological dimension: Refers to an individual’s knowledge of COPD diagnostic guidelines, the perceived value of early diagnosis, and psychological readiness to initiate diagnostic behaviors. This dimension is measured via the COPD Diagnostic Adherence Literacy Scale, which captures three subcomponents: (a) disease-specific knowledge; (b) perceived benefit; (c) psychological readiness.
- Behavioral dimension: Refers to the completion of evidence-based diagnostic procedures as outlined in the Chinese Guidelines for COPD Diagnosis and Management (2021), specifically: (a) post-bronchodilator spirometry; (b) chest imaging (X-ray or CT) to exclude alternative diagnoses; and (c) formal diagnosis documented by a qualified pulmonologist. This dimension is quantified as the diagnostic coverage rate.

This dual-dimensional framework aligns with WHO recommendations for measuring adherence in chronic diseases, which emphasize the need to integrate “intentional non-adherence” (driven by cognitive barriers) and “unintentional non-adherence” (driven by contextual barriers).

### 1.2. Theoretical Framework: The Health Belief Model

The Health Belief Model (HBM) is a widely validated social cognitive framework for explaining health-seeking behaviors, with six core constructs: (1) perceived susceptibility; (2) perceived severity; (3) perceived benefits; (4) perceived barriers; (5) self-efficacy; (6) cues to action [14]. Since its development in the 1950s, the HBM has been applied to predict a range of COPD-related behaviors, including smoking cessation, inhaler adherence (β = 0.28 for perceived benefits) and pulmonary rehabilitation participation (β = 0.31 for perceived severity).

However, the utility of the HBM in explaining COPD diagnostic adherence in rural China remains underexplored. Most prior HBM-based COPD studies have been conducted in high-income countries (HICs) [14], where findings may not generalize to rural LMIC settings. Most prior HBM-based COPD studies have been conducted in high-income countries, where perceived benefits have been identified as an important predictor of screening behavior [15]; however, these findings may not generalize to rural LMIC settings with unique contextual barriers such as indirect healthcare costs and limited trust in medical institutions [16]. A qualitative study in rural Henan (China) found that 62% of COPD high-risk individuals reported “perceived barriers (time/cost) outweigh perceived benefits”—even among those with high perceived susceptibility. This suggests that the HBM’s traditional constructs may need to be supplemented with context-specific variables to explain adherence in rural LMICs.

Recent adaptations of the HBM in LMICs have integrated two critical contextual factors: (1) social support (instrumental support: practical assistance such as transport; emotional support: encouragement from family/friends) [17]; (2) economic toxicity (financial burden of healthcare, including direct costs [copayments, medications] and indirect costs [transport, lost wages]) [18,19]. In rural China, these factors are particularly salient: 79% of rural residents have monthly incomes ≤ 2000 RMB [20], and even with basic medical insurance, out-of-pocket costs for COPD screening and diagnostic procedures can represent a substantial proportion of monthly income for rural residents [21]. Additionally, 15.3% of rural elderly adults live alone [22], and social isolation has been linked to a 40% higher risk of non-adherence to chronic disease care. Despite this, few studies have quantitatively tested the mediating or moderating roles of social support and economic toxicity within the HBM framework for COPD diagnostic adherence.

### 1.3. Research Gaps and Objectives

A comprehensive review of the literature identifies four critical gaps that limit our understanding of COPD diagnostic adherence in rural China:

(1) Overreliance on single-dimensional behavioral measures: Most studies assess diagnostic adherence solely through behavioral outcomes, ignoring cognitive barriers that precede behavioral inaction. Without measuring cognitive adherence, interventions may fail to address the root causes of non-adherence.
(2) Inadequate quantification of economic toxicity and social support: Studies rarely distinguish between direct and indirect costs of screening, and social support is often measured as a single construct despite its differential effects on adherence dimensions.
(3) Untested roles of health beliefs and social support: The pathways through which COPD knowledge and economic status influence adherence remain unclear, which limits the design of targeted interventions.
(4) Neglect of anxiety as a potential confounder: Anxiety is prevalent in COPD populations and has been associated with healthcare-seeking behavior across chronic conditions, but it has rarely been included in models of adherence in COPD [23,24].

To address these gaps, this study has three primary objectives:

(1) Identify demographic, cognitive, and contextual predictors of two-dimensional diagnostic adherence in rural Chinese COPD high-risk populations, with anxiety included as a potential confounder.
(2) Quantify the mediating effects of health beliefs and social support on the relationships between (a) COPD knowledge and diagnostic adherence and (b) economic toxicity and diagnostic adherence.
(3) Develop evidence-based, resource-appropriate primary care interventions to improve diagnostic adherence that are tailored to the unique needs of rural Chinese populations.

## 2. Materials and Methods

### 2.1. Study Design and Setting

A cross-sectional survey was conducted from 17 to 22 March 2025 in Pingdu District, Qingdao City, Shandong Province—an eastern Chinese district with a total population of 1.38 million. Pingdu was selected as the study site for three reasons: (1) its demographic and socioeconomic characteristics are broadly comparable to those of other rural districts in eastern China; (2) its healthcare system mirrors the national rural structure: 21 township health centers, 8 secondary/higher-level hospitals; (3) prior data from Pingdu CDC show a COPD prevalence of 14.2% among adults ≥ 40 years—consistent with the national rural prevalence [25]. A two-stage screening and diagnostic procedure was employed. In Stage 1 (screening), research staff administered the questionnaire and conducted portable pre-bronchodilator spirometry at township health centers to identify eligible high-risk individuals. In Stage 2 (diagnostic evaluation), eligible participants were referred to secondary or higher-level hospitals for standard post-bronchodilator spirometry, chest imaging, and specialist consultation. The behavioral adherence outcome was defined based on completion of Stage 2 diagnostic procedures, which are distinct from the Stage 1 screening spirometry.

The study protocol was approved by the Institutional Review Board of School of Public Health, Shandong University (Approval No. IRB-SPH-SDU-20250362) and conducted in accordance with the Declaration of Helsinki (2023 Revision) [22]. All participants provided written informed consent; for participants with limited literacy, verbal consent was obtained in the presence of a family member or village health worker (VHW), with consent recorded via audio and witnessed signature.

### 2.2. Eligibility Criteria

#### 2.2.1. Inclusion Criteria

The inclusion criteria were based on the Chinese Guidelines for COPD Diagnosis and Management (2021) [23] and previous studies of high-risk COPD populations:

Aged ≥ 40 years.

At least one COPD high-risk factor: (a) smoking history ≥ 10 pack-years; (b) first-degree relative with COPD; (c) chronic cough/sputum production for ≥3 months/year over 2 consecutive years; (d) long-term biomass fuel exposure or occupational exposure to dust/pollutants.

Positive first-stage screening: pre-bronchodilator FEV1/FVC < 0.70 on portable spirometry conducted at township health centers during the screening process. Note: This screening spirometry is distinct from the standard post-bronchodilator spirometry required for formal COPD diagnosis at secondary or higher-level hospitals (see Section 2.1). The FEV1/FVC criterion was used alone (rather than FEV1 < 80% predicted) because post-bronchodilator FEV1/FVC < 0.70 is the established pulmonary-function criterion for persistent airflow limitation per the Chinese COPD guideline [24]; FEV1 < 80% predicted alone may occur in restrictive conditions and does not establish airflow obstruction.

Ability to communicate proficiently in Mandarin or the Pingdu dialect.

Resident of Pingdu District for ≥6 months.

#### 2.2.2. Exclusion Criteria

Severe cognitive impairment (MMSE score < 24) [25].

Severe psychiatric disorders were diagnosed per medical records.

End-stage chronic diseases with life expectancy < 6 months.

Physical disability limiting the ability to access hospitals.

Previous formal COPD diagnosis.

Refusal to participate or to provide consent.

### 2.3. Sampling Strategy

Stratified random sampling was used to enhance sample diversity across geographic and socioeconomic strata, with three hierarchical layers:

Layer 1: Geographic Stratification

Pingdu’s 21 township health centers were stratified into four geographic layers: remote rural, suburban, coastal rural, and mountainous rural.

Layer 2: Health Center and Village Selection

From each geographic layer, 1 township health center was randomly selected. For each selected health center, 2 administrative villages were randomly selected, stratified by population size.

Layer 3: Individual Selection

Ultimately, eight participants were replaced, yielding a final sample of 248. The response rate among invited eligible individuals was 92.5% (248/268). An a priori sample size calculation was conducted using G*Power 3.1 for multiple linear regression, assuming a medium effect size (f^2^ = 0.15), α = 0.05, power = 0.80, and up to 10 predictor variables. The minimum required sample size was 167. Accounting for an estimated 20% non-response or ineligibility, a target sample of 200 was set; the final sample of 248 exceeded this target. Given the clustered nature of sampling (4 health centers, 8 villages), cluster-robust standard errors were employed as a sensitivity analysis; because the number of clusters was small, results from the standard analysis are presented as primary, with cluster-robust results noted where they differed materially.

### 2.4. Data Collection Tools

All data collection tools were either validated for rural Chinese populations or developed and validated specifically for this study. Tools were pilot-tested in a non-study village (n = 30) with minor wording revisions.

#### 2.4.1. General Demographic and Clinical Questionnaire

A self-designed questionnaire with 23 items was used to collect sociodemographic (gender, age, education, income, insurance, transport) and clinical (family history, comorbidities, exposure, health education) data. The content validity index (CVI) was 0.89.

#### 2.4.2. Measures of Diagnostic Adherence

Cognitive/Psychological Dimension: COPD Diagnostic Adherence Literacy Scale.

This scale was developed for this study with 9 items (3 per domain: disease knowledge, perceived benefit, psychological readiness), scored 0–9 (higher = better cognitive adherence). Full item content is provided in Supplementary Table S3. The scale development procedure included: (1) item generation via literature review and semi-structured interviews with 12 COPD patients and 5 clinicians, yielding 20 initial items; (2) expert review by a panel of 5 experts (2 pulmonologists, 2 public health researchers, 1 nurse specialist), with item-level content validity index (I-CVI) ≥ 0.80 and scale-level CVI (S-CVI) = 0.92; (3) pilot testing in 30 participants from a non-study village, leading to minor wording revisions; (4) exploratory factor analysis (EFA) in a randomly split half-sample (n = 124), which extracted three factors explaining 68.4% of variance; (5) confirmatory factor analysis (CFA) in the independent second half-sample (n = 124), confirming the three-factor structure. Test–retest reliability was assessed in 30 participants at a 2-week interval (ICC = 0.89, two-way random effects model, absolute agreement).

Behavioral Dimension: Diagnostic Coverage Rate.

This outcome was defined as the completion of standard diagnostic procedures at secondary or higher-level hospitals during Stage 2 evaluation. Three steps were assessed: (a) standard post-bronchodilator spirometry (essential for COPD diagnosis per Chinese guideline [26]); (b) chest imaging (X-ray or CT), recommended by the guideline for differential diagnosis and complication assessment; (c) formal diagnosis documented by a qualified pulmonologist. The primary behavioral outcome was binary: completion of all three steps (complete diagnostic completion) versus not. A graded measure (0–3 steps completed) was also computed and used in sensitivity analyses. The requirement of specialist diagnosis was adopted as a study-specific criterion to ensure diagnostic accuracy; we acknowledge that this may partly reflect access to specialist care rather than patient behavior alone, and this limitation is addressed in the Discussion. Data were extracted from hospital medical records by two independent researchers, with high inter-rater reliability (κ = 0.91).

#### 2.4.3. Validated Scales for Predictor and Confounder Variables

General health literacy was measured using the European Health Literacy Survey Questionnaire-Short Form (HLS-EU-Q16) a 16-item instrument assessing health literacy across healthcare, disease prevention, and health promotion domains. Each item is rated on a 4-point scale (1 = very difficult to 4 = very easy), with total scores ranging from 16 to 64 (higher scores indicate better health literacy). The HLS-EU-Q16 has been validated in Chinese populations and, in the present sample, demonstrated excellent internal consistency (Cronbach’s α = 0.854) and test–retest reliability (ICC = 0.88). All scales were previously validated in Chinese populations with minor adaptations for rural contexts (Table 1).

### 2.5. Data Collection Procedures

Data collection was conducted by 10 trained research staff over 6 days and followed four steps:

Research staff training: A 2-day training program covered the study protocol, questionnaire administration, medical record review, and ethical requirements. Staff with ICC < 0.85 received additional training.

Face-to-face interviews: Interviews were conducted in private village clinic or community center spaces and lasted 25–35 min. For low-literacy participants, questionnaires were administered orally in the local dialect. Researchers were blinded to participants’ spirometry results.

Medical record review: Diagnostic data were extracted from eight secondary/higher hospitals using a standardized form. Missing records were supplemented via follow-up calls.

Quality control: In total, 10% of questionnaires and 15% of medical records were randomly rechecked for accuracy. No missing data were observed in the final dataset.

### 2.6. Statistical Analysis

All statistical analyses were performed using SPSS 27.0, Python 3.9, and R 4.3.1 (lavaan package for CFA) with a two-tailed significance level of α = 0.05. The analysis followed the STROBE guidelines.

Descriptive statistics: Categorical variables presented as frequencies and percentages, and continuous variables as mean ± SD, median, and interquartile range.

Reliability and validity testing: Cronbach’s α and ICC for reliability, CVI/CVR for content validity, and EFA/CFA for construct validity.

Group comparisons: For the continuous cognitive outcome, independent samples *t*-tests and one-way ANOVA were used, with Cohen’s d and η^2^ as effect sizes. For the binary behavioral outcome, chi-square tests (or Fisher’s exact test when expected cell counts were <5) were used, with odds ratios and 95% confidence intervals as effect measures. All *p*-values from group comparisons and correlation analyses were adjusted for multiple comparisons using the Benjamini–Hochberg (BH) false discovery rate (FDR) procedure. The BH procedure was applied separately to two pre-specified families: (a) all group-comparison tests reported in Table 2 (14 tests comprising 7 cognitive and 7 behavioral comparisons); and (b) all pairwise correlation tests reported in Table 3 (36 unique pairs). Both raw and FDR-adjusted *p*-values are reported in the tables.

Correlation analysis: Pearson correlation coefficients were used for associations between continuous variables (including the cognitive outcome). Point-biserial correlations were used for associations involving the binary behavioral outcome. FDR correction was applied.

Regression analysis: For the continuous cognitive outcome, stepwise multiple linear regression was used to identify independent predictors, with collinearity diagnostics (variance inflation factor, VIF), the Durbin–Watson statistic, and residual analyses reported. For the binary behavioral outcome, multivariable logistic regression was employed, reporting odds ratios (ORs) with 95% confidence intervals [30]. Model fit for logistic regression was assessed using the Hosmer–Lemeshow test, Nagelkerke R^2^, and the area under the receiver operating characteristic curve (AUC). A linear probability model with heteroskedasticity-robust standard errors was run as a sensitivity analysis for the behavioral outcome and produced consistent results [31].

Mediating effect analysis: Hayes’ PROCESS macro (Model 4) with 5000 bootstrap samples was used to test the mediating effects of health beliefs and social support, controlling for age, education, and anxiety.

## 3. Results

### 3.1. Participant Characteristics

#### 3.1.1. Sociodemographic Characteristics

The sample included 131 females (52.82%) and 117 males (47.18%), with a mean age of 68.2 ± 8.7 years. Education level was generally low: 14.92% had no formal education, 38.31% had primary school, and only 2.42% had college or higher; 79.44% of participants had a monthly family income ≤ 2000 RMB, and 90.73% were covered by the Rural Cooperative Medical Scheme; 49.19% lived > 30 min from the nearest secondary/higher hospital, and 39.11% lived alone. All characteristics of participants can be seen at Table 4.

#### 3.1.2. Clinical Characteristics and COPD Risk Factors

In total, 26.61% were current smokers, 28.23% former smokers, and 45.16% never smokers; 86.69% had at least one first-degree relative with COPDl; 38.71% had long-term biomass fuel exposure, and 15.32% had occupational dust/pollutant exposure; 66.53% had at least one comorbidity (165/248), with hypertension being the most common (53.23%). Only 12.10% had participated in COPD health education programs. All characteristics o be seen at Table 5.

### 3.2. Reliability and Validity of Measurement Tools

All scales demonstrated acceptable to excellent levels of reliability and validity. Cronbach’s α coefficients ranged from 0.710 (COPD-Q) to 0.892 (EQ-5D-5L), all exceeding the 0.70 threshold. Test–retest ICC values ranged from 0.86 to 0.90, indicating good stability [32].

Content validity indices for all tools exceeded 0.80. For the newly developed COPD Diagnostic Adherence Literacy Scale, EFA was conducted in the first random half-sample (n = 124). The KMO measure was 0.798 and Bartlett’s test of sphericity was significant (χ^2^ = 300.329, df = 36, *p* < 0.001), supporting factorability. Principal axis factoring with promax rotation extracted three factors with eigenvalues > 1.0, explaining 68.4% of total variance. Factor loadings ranged from 0.62 to 0.85, with no cross-loadings > 0.40. CFA was conducted in the independent second half-sample (n = 124) to verify the three-factor structure. The model demonstrated acceptable fit: χ^2^/df = 1.87, CFI = 0.958, TLI = 0.943, RMSEA = 0.059 (90%CI: 0.041–0.078), SRMR = 0.048. All factor loadings were statistically significant (*p* < 0.001) and ranged from 0.58 to 0.83. To examine whether cognitive and behavioral adherence represent distinct constructs, a CFA comparing a one-factor model (all adherence items loading on a single factor) versus a two-factor model (cognitive and behavioral as separate but correlated factors) was conducted. The two-factor model showed superior fit: χ^2^/df = 2.34, CFI = 0.942, TLI = 0.928, RMSEA = 0.074 (90%CI: 0.061–0.088), SRMR = 0.052, compared with the one-factor model: χ^2^/df = 4.12, CFI = 0.856, TLI = 0.831, RMSEA = 0.112 (90%CI: 0.098–0.127), SRMR = 0.089. The chi-square difference test was significant (Δχ^2^ = 156.3, Δdf = 1, *p* < 0.001), supporting the two-factor structure. However, the inter-factor correlation was high (r = 0.78), indicating substantial overlap; this is acknowledged as a limitation. Discriminant validity was assessed by comparing the square root of the average variance extracted (AVE) for each construct with its inter-construct correlations. The cognitive adherence scale had AVE = 0.58 (sqrt = 0.76); its correlations with COPD knowledge (r = 0.786) and HBM total score (r = 0.788) slightly exceeded the sqrt(AVE), suggesting some degree of construct overlap, which is addressed in the Discussion. Convergent validity was supported by significant correlations in theoretically expected directions with all external constructs. The Reliability and validity can be seen at Table 6.

### 3.3. Descriptive Statistics of Key Variables

Cognitive adherence had a mean score of 4.935 ± 2.512, with 32.66% classified as low adherence (score ≤ 3). The behavioral diagnostic coverage rate was 42.3%; among non-adherent participants, 31.05% failed to complete spirometry, 28.67% lacked chest imaging, and 40.28% had no official diagnosis [33].

The mean COPD knowledge score was 7.181 ± 3.367, with only 28.63% scoring ≥10. Mean health literacy score (HLS-EU-Q16) was 32.47 ± 8.62 (range: 16–58), with 43.2% classified as having inadequate or problematic health literacy (score < 33). Mean total HBM score was 107.157 ± 31.803, with perceived barriers being the lowest dimension. Mean social support score was 24.387 ± 8.872, and mean economic toxicity score was 4.948 ± 2.644, with 41.53% reporting severe economic toxicity. Mean anxiety score was 4.21 ± 2.89, with 12.10% meeting probable anxiety criteria. All descriptive statistics can be seen at Table 7.

### 3.4. Group Differences in Diagnostic Adherence

Significant group differences were observed across multiple demographic and clinical variables:

Gender: Females showed higher cognitive and behavioral adherence than males (d = 0.64).

Medical accessibility: Participants living ≤ 30 min from the hospital had higher adherence (d = 0.59).

Living status: Participants living with a spouse/children had the highest adherence, while those living alone had the lowest (η^2^ = 0.42).

Comorbidities and family history: Participants with comorbidities or a family history of COPD had significantly higher adherence.

Smoking status: Former smokers had the highest cognitive and behavioral adherence, followed by never smokers and current smokers (η^2^ = 0.11 for cognitive; chi-square = 8.92, *p* = 0.012 for behavioral).

Anxiety: Participants with probable anxiety showed lower adherence (d = 0.49).

Age, education level, and comorbidity count showed no significant association with either adherence dimension.

### 3.5. Correlation Analysis

Pearson correlation analysis revealed strong, theoretically consistent relationships:

Cognitive adherence showed strong positive correlations with COPD knowledge (r = 0.786), health beliefs (r = 0.788), social support (r = 0.723), and health literacy (r = 0.731), and a strong negative correlation with economic toxicity (r = −0.630).

Behavioral adherence showed strong positive correlations with health literacy (r = 0.715), social support (r = 0.701), and COPD knowledge (r = 0.752), and a strong negative correlation with economic toxicity (r = −0.602).

Anxiety was weakly correlated with both adherence dimensions.

Health beliefs were strongly correlated with COPD knowledge (r = 0.764), supporting the theoretical proposition of the HBM.

Additional correlation analyses were conducted for HBM sub-dimensions and economic toxicity subcomponents. Perceived benefits showed a strong positive correlation with cognitive adherence (r = 0.68, *p* < 0.001), and self-efficacy showed a moderate-to-strong positive correlation (r = 0.61, *p* < 0.001). For economic toxicity, indirect cost concerns (transport, lost wages, accompaniment time) were more strongly negatively correlated with behavioral adherence (r = −0.52, *p* < 0.001) than direct cost concerns (copayments, procedure fees) (r = −0.38, *p* < 0.001). A gender-by-living-status interaction analysis was conducted for both adherence dimensions; the interaction term was not statistically significant for either cognitive (*p* = 0.12) or behavioral (*p* = 0.18) adherence, although the descriptive pattern suggested a stronger negative association of living alone among men than women. All *p*-values in this supplementary analysis were FDR-adjusted within the correlation family (36 unique pairs plus these supplementary tests).

### 3.6. Multiple Regression Analysis

#### 3.6.1. Cognitive Adherence Regression Model (Linear Regression)

The final model included 7 predictors, explaining 63.4% of the variance (R^2^ = 0.634, Adjusted R^2^ = 0.623, F = 59.355, df = 7/240, *p* < 0.001). The strongest predictors were health literacy (β = 0.286), health beliefs (β = 0.252), and COPD knowledge (β = 0.239). Negative predictors included living alone (β = −0.157) and economic toxicity (β = −0.108). Comorbidities and COPD family history were weak positive predictors. Anxiety, age, and education did not enter the model. Regression diagnostics indicated no multicollinearity concerns (all VIF < 2.5, mean VIF = 1.72), no significant autocorrelation (Durbin–Watson = 1.98), and approximately normally distributed residuals (Shapiro–Wilk *p* = 0.087). One participant was identified as a potential outlier (Cook’s distance = 0.08 < 1.0); analyses with and without this participant produced consistent results.

#### 3.6.2. Behavioral Adherence Regression Model (Logistic Regression)

The final multivariable logistic regression model included 4 predictors (Table 8). Model fit was adequate: Hosmer–Lemeshow χ^2^ = 7.23, df = 8, *p* = 0.512; Nagelkerke R^2^ = 0.68; AUC = 0.89 (95%CI: 0.85–0.93). Predictors of complete behavioral adherence were: health literacy (OR = 1.28, 95%CI: 1.15–1.42, *p* < 0.001, per 1-point increase in HLS-EU-Q16), social support (OR = 1.12, 95%CI: 1.04–1.21, *p* = 0.005, per 1-point increase in mMOS-SS), economic toxicity (OR = 0.82, 95%CI: 0.72–0.94, *p* = 0.004, per 1-point increase), and medical accessibility (OR = 1.85, 95%CI: 1.12–3.05, *p* = 0.016, ≤30 vs. >30 min). Health beliefs, anxiety, and smoking status did not enter the model. A sensitivity analysis using a linear probability model with heteroskedasticity-robust standard errors yielded directionally consistent results. VIF values for all predictors were <2.0, indicating no multicollinearity.

#### 3.6.3. Sensitivity Analysis: Construct Overlap

Given the high correlations between the cognitive adherence scale and COPD knowledge (r = 0.786) and HBM total score (r = 0.788)—which exceeded the square root of AVE (0.76) and indicated construct overlap—a sensitivity analysis was conducted using a reduced cognitive adherence score that excluded the three disease-knowledge items and the three perceived-benefit items, retaining only the three psychological-readiness items (range 0–3). This reduced score had a mean of 1.7 ± 1.18 and acceptable internal consistency (Cronbach’s α = 0.74). A multiple linear regression with the same seven predictors explained 41.2% of the variance (Adjusted R^2^ = 0.395, F = 24.18, df = 7/240, *p* < 0.001). Health literacy (β = 0.241, *p* < 0.001), social support (β = 0.198, *p* < 0.001), and living alone (β = −0.172, *p* < 0.001) remained significant independent predictors. In contrast, COPD knowledge (β = 0.089, *p* = 0.12) and health beliefs (β = 0.103, *p* = 0.08) were no longer statistically significant, confirming that the overlap was driven primarily by the knowledge and perceived-benefit subcomponents of the cognitive adherence scale [34]. These findings indicate that the significant associations of COPD knowledge and health beliefs with the full cognitive adherence score should be interpreted cautiously, as they partly reflect item-level content overlap rather than fully independent predictive effects.

### 3.7. Mediating Effect Analysis

#### 3.7.1. Statistical Mediation Path 1: COPD Knowledge → Adherence

Health beliefs as mediator: A statistically significant indirect association was observed for both dimensions. For cognitive adherence, health beliefs accounted for 31.4% of the total association between COPD knowledge and cognitive adherence. For the behavioral outcome, the indirect association was estimated on the log-odds scale; the proportion mediated (28.7%) was computed as ab/c on the log-odds metric, following the approach described by Hayes (2013) for logistic outcomes in PROCESS Model 4, in which all paths—including the outcome path—are estimated on the log-odds scale and the indirect effect, ab, is the product of the log-odds coefficients. This proportion should be interpreted strictly on the log-odds scale and not as a proportion of the probability-scale effect, owing to the non-collapsibility of odds ratios [35].

Social support as mediator: The indirect association via social support was not statistically significant for cognitive adherence after FDR correction (12.0%, bootstrap 95%CI [−0.007, 0.162], *p* = 0.068) but was significant for behavioral adherence (18.5%, bootstrap 95%CI [0.15, 2.49], *p* = 0.027).

#### 3.7.2. Statistical Mediation Path 2: Economic Toxicity → Adherence

Health beliefs as mediator: A statistically significant indirect association was observed for both dimensions. For cognitive adherence, health beliefs accounted for 36.9% of the total negative association between economic toxicity and cognitive adherence; for behavioral adherence (log-odds scale), they accounted for 41.2% [36].

Social support as a mediator: The indirect associations via social support were not statistically significant after FDR correction for either dimension (cognitive: 14.8%, *p* = 0.062; behavioral: 20.3%, *p* = 0.065). E-values for the significant indirect pathways ranged from 1.82 to 2.35. The E-value quantifies the minimum strength of association that an unmeasured confounder would need to have with both the exposure and the outcome (on the risk-ratio scale) to explain away the observed association, conditional on the measured covariates [37,38]. An E-value of 1.8–2.4 indicates that a confounder would need to be associated with both the exposure and mediator at a relative risk of approximately 1.8–2.4 or greater to attenuate the indirect association to the null. All mediation results should be interpreted as exploratory statistical associations, not causal effects, given the cross-sectional design. The complete bootstrap-mediated effect tests, including total, direct, and indirect effects with bias-corrected 95% bootstrap confidence intervals and FDR-adjusted *p*-values for both exposure pathways, are summarized in Table 9.

## 4. Discussion

This study is among the first to systematically investigate two-dimensional COPD diagnostic adherence (cognitive/behavioral) in rural Chinese high-risk populations using the HBM, with three key contributions: (1) identifying modifiable factors associated with adherence across cognitive and contextual domains; (2) exploring the statistical mediating roles of health beliefs and social support [41]; and (3) proposing resource-appropriate primary care intervention strategies for future prospective evaluation. The dual-dimensional conceptualization of adherence—encompassing both cognitive/psychological readiness and behavioral completion—has precedents in other chronic disease studies, including diabetes medication adherence, hypertension care engagement, and cancer screening behavior, where cognitive antecedents and behavioral outcomes have been shown to be related but distinct constructs.

### 4.1. Alignment with Global Evidence and Rural Specificity

#### 4.1.1. Health Literacy: The Strongest Modifiable Predictor

Health literacy emerged as the strongest modifiable factor associated with both adherence dimensions, consistent with global studies showing that health literacy is associated with chronic disease care engagement. In the present sample, 43.2% of participants had inadequate or problematic health literacy (HLS-EU-Q16 score < 33), and many held misconceptions about COPD diagnosis—for example, believing that a chest X-ray alone is sufficient for diagnosis without spirometry [42]. A unique rural challenge is the potential information gap via village health workers (VHWs), who serve as the primary point of contact for many rural residents. While formal VHW training status was not systematically measured in this study, qualitative feedback from field staff suggested that COPD-specific training among VHWs may be limited. Improving VHW COPD training is a promising strategy for bridging the health literacy gap in rural areas, though this requires formal evaluation [43].

#### 4.1.2. Health Beliefs: Validating the HBM in Rural China

Health beliefs were associated with cognitive adherence and statistically mediated a substantial proportion of the associations between knowledge, economic toxicity, and cognitive adherence (31.4% and 36.9%, respectively). For the behavioral outcome, indirect associations were observed on the log-odds scale (28.7% and 41.2%). These findings are broadly consistent with the HBM’s theoretical propositions in rural LMIC settings, although the high construct overlap between cognitive adherence, COPD knowledge, and HBM scores (see Section 3.2 and Limitations) means that these mediating pathways should be interpreted as exploratory statistical associations rather than fully independent causal pathways. Three HBM dimensions warrant rural-specific attention:

Perceived barriers: The lowest HBM dimension score reflected two rural-specific concerns: the time cost of work absence and distrust in hospital diagnoses. In HICs, barriers typically focus on direct costs, but rural China’s informal economy means time off work translates directly to lost income.

Perceived benefits: Perceived benefit scores were positively correlated with cognitive adherence (r = 0.68, *p* < 0.001), a pattern consistent with urban studies, though potentially amplified in rural settings where community testimonials carry greater weight.

Self-efficacy: Self-efficacy in arranging transport and clinic visits was positively correlated with cognitive adherence (r = 0.61, *p* < 0.001), as rural logistical barriers make practical capability more critical than it is in urban areas.

#### 4.1.3. Economic Toxicity: Persistent Barriers Despite Insurance Coverage

Economic toxicity was negatively associated with both adherence dimensions, even with 90.7% insurance coverage. Rural China’s indirect costs are particularly burdensome: out-of-pocket screening costs represented approximately 7.5% of average monthly income, and qualitative feedback indicated that travel time and potential lost wages were frequently cited as reasons for deferring diagnostic visits. Financial stress also appeared to distort health beliefs: among participants with severe economic toxicity, a substantial proportion agreed that “screening is useless if I can’t afford treatment”—a cognitive barrier less commonly observed in insured urban populations. These findings underscore that insurance coverage alone may be insufficient without addressing indirect costs and perceived financial barriers [44].

#### 4.1.4. Social Support: Instrumental Support Drives Behavioral Adherence

Social support was associated with both adherence dimensions. A rural-specific finding was that instrumental support (transport, accompaniment) correlated more strongly with behavioral adherence than emotional support [45], reflecting rural practical needs: 49.19% lived >30 min from a hospital, and 39.11% lived alone, making transport and accompaniment critical for completing diagnostic tests.

#### 4.1.5. Anxiety: Weak Correlation, Non-Significant Predictor

Anxiety was weakly correlated with adherence but was not an independent predictor. Three factors may explain this: rural stigma around mental health leading to potential underdiagnosis [46], the small anxiety subgroup size, and social support partially mediating anxiety’s effect on adherence.

### 4.2. Advancing Understanding of Rural COPD Diagnostic Adherence

This study addresses four critical gaps in the literature:

Dual-dimensional adherence measurement: By including cognitive adherence, we found that 32.66% of participants had low cognitive readiness even though 42.3% completed behavioral diagnostic steps. This “cognitive-behavioral mismatch” has not been reported in rural COPD studies, and such participants may be less likely to engage in long-term management.

Mediating mechanisms of health beliefs: We quantified that health beliefs mediate 31.4% of the knowledge → cognitive adherence effect and 41.2% of the economic toxicity → behavioral adherence effect. No prior study has quantified these pathways in rural China.

Indirect costs as a key barrier: We distinguished between direct costs (copayments, procedure fees) and indirect costs (transport, lost wages, accompaniment time) using the Financial Hardship Scale and found that indirect cost concerns were more strongly associated with non-adherence than direct cost concerns (r = −0.52 vs. r = −0.38, both *p* < 0.001). This provides a more accurate picture of rural financial barriers than HIC studies focusing only on direct costs.

Gender-specific social isolation effects: Living alone was associated with significantly lower adherence for both men and women; however, the negative association was more pronounced among men living alone than among women living alone; a formal gender-by-living-status interaction test was not statistically significant (*p* = 0.12), and this pattern should therefore be interpreted as descriptive. This pattern may reflect gender differences in community engagement and social network density, and has implications for tailored interventions.

### 4.3. Clinical Implications: Tailored Interventions for Rural Primary Care

Based on the present cross-sectional findings, we propose a three-tiered intervention framework aligned with the Care Cascade Model and designed for rural resource-constrained settings. These strategies are proposed as future directions requiring prospective evaluation (e.g., randomized controlled trials or pragmatic implementation studies), as the present cross-sectional design cannot establish intervention efficacy. The framework is organized by the level at which barriers operate:

Tier 1: Cognitive-Level Interventions

Develop picture-text COPD educational materials in the local dialect, focusing on spirometry as the diagnostic gold standard, insurance coverage policies, and local success stories. Provide 8 h COPD training to VHWs with quarterly refresher courses, enabling monthly home visits to high-risk individuals. Host monthly “COPD Screening Days” in village clinics, including spirometry demonstrations, role-playing exercises, and peer testimonials from early-diagnosed residents. Address perceived barriers by scheduling workshops on weekends, displaying hospital accreditation certificates, and providing basic amenities to increase attendance.

Tier 2: Behavioral-Level Interventions

Advocate for enhanced insurance coverage for COPD screening and diagnostic procedures, as economic toxicity has been identified as a consistent barrier even with existing insurance coverage. Policy efforts to reduce out-of-pocket costs and cover indirect expenses (transport, lost wages) should be explored and evaluated in prospective studies. Partner with local organizations to provide transport vouchers and meal allowances for screening visits. Establish monthly mobile screening units equipped with spirometers for remote rural villages. Train township health center staff to conduct basic spirometry, enabling “one-stop” screening at local health centers.

Tier 3: Social-Level Interventions

Establish “COPD Buddy Systems”: Pair isolated older adults (especially males) with community buddies who provide transport, accompaniment, and post-screening follow-up, with small grocery vouchers as incentives. Organize gender-specific social groups: monthly men’s health clubs combining COPD education with social activities, and integration of COPD education into existing village women’s groups.

### 4.4. Strengths and Limitations

#### 4.4.1. Strengths

The dual-dimensional adherence model captures both cognitive and behavioral barriers, addressing a key literature gap. Stratified random sampling across four geographic strata enhanced sample diversity; medical record verification reduced recall bias; validated scales enhanced reliability. Exploring health beliefs and social support as statistical mediators provides hypothesized pathways that warrant future longitudinal testing. The study focuses on the understudied rural population that bears a disproportionate share of China’s COPD burden.

#### 4.4.2. Limitations

Several limitations should be acknowledged. First, the cross-sectional design cannot establish causality or temporal ordering; all mediation analyses should be interpreted as exploratory statistical indirect associations, and reverse causality is possible (e.g., individuals who have completed diagnostic testing may subsequently acquire more COPD knowledge). Longitudinal studies are needed to confirm directional effects. Second, findings are from one district in eastern China and may not generalize to remote western China with poorer healthcare access, or to urban populations. Third, the behavioral adherence composite—requiring specialist diagnosis by a pulmonologist—may partly reflect access to specialist care rather than patient behavior alone; this could inflate associations with structural factors such as medical accessibility. Fourth, the cognitive adherence scale showed substantial construct overlap with COPD knowledge (r = 0.786) and HBM constructs (r = 0.788), with the square root of AVE for cognitive adherence (0.76) lower than its correlation with these external constructs. This overlap substantially limits the interpretation of COPD knowledge and health beliefs as fully independent predictors of cognitive adherence. A sensitivity analysis using a reduced cognitive adherence score that excluded the disease-knowledge and perceived-benefit items (retaining only psychological-readiness items, range 0–3) found that health literacy, social support, and living alone remained significant predictors, whereas COPD knowledge and health beliefs were no longer significant (see Section 3.6.3), confirming that the overlap was driven primarily by the knowledge and perceived-benefit subcomponents. While the CFA supported a two-factor structure over a one-factor structure, the high inter-factor correlation (r = 0.78) indicates that cognitive and behavioral adherence are closely related. Future research should further refine the cognitive adherence measure to improve discriminant validity. Fifth, doctor–patient communication quality and VHW training status were not formally measured. Sixth, rural stigma around mental health may have led to underreporting of anxiety symptoms. Seventh, the sample size of 248, while exceeding the a priori calculation, is modest for psychometric scale development and multilevel modeling; the small number of clusters (4 health centers, 8 villages) limited our ability to conduct robust multilevel analyses, and cluster-robust standard errors were used as a sensitivity analysis instead. Finally, the graded behavioral adherence measure (0–3 steps) was used only in sensitivity analyses; future studies may benefit from treating partial adherence as the primary outcome to capture more nuanced patterns.

## 5. Conclusions

In this cross-sectional study of rural COPD high-risk populations in eastern China, diagnostic adherence was associated with a complex interplay of cognitive factors (health literacy, health beliefs, COPD knowledge) and contextual factors (social support, economic toxicity, medical accessibility, living status). Health literacy emerged as the strongest modifiable factor associated with adherence, while health beliefs statistically mediated the associations between knowledge, economic status, and cognitive adherence. Key rural-specific insights include the primacy of instrumental social support, the burden of indirect costs alongside direct costs, and gender differences in the impact of social isolation. Given the cross-sectional design, these findings should be interpreted as associations rather than causal effects.

Interventions to improve diagnostic adherence should adopt a dual-target approach: (1) cognitive-level strategies to improve health literacy and strengthen health beliefs; (2) behavioral-level strategies to reduce financial barriers and optimize accessibility; and (3) social-level strategies to enhance support networks, especially for isolated males. These intervention strategies are proposed as future directions requiring prospective evaluation. The present findings provide a preliminary evidence base for optimizing early COPD diagnosis in rural primary care and offer actionable insights for other LMICs facing similar healthcare challenges.

Future research should use longitudinal designs to confirm causal relationships and temporal ordering, test the proposed intervention framework in randomized controlled trials or pragmatic implementation studies, expand to remote western China to assess generalizability, include objective anxiety measures and assessments of doctor–patient communication, and further refine the cognitive adherence measure to improve discriminant validity from related constructs. Multilevel modeling with larger numbers of clusters would also strengthen future investigations.

## Figures and Tables

**Table 1:** Predictor and confounder variables.

Scale Name	Purpose	Number of Items	Scoring Range	Psychometric Properties
COPD Knowledge Questionnaire (COPD-Q)	Assess COPD-related knowledge	13	0–13	Cronbach’s α = 0.710; test–retest ICC = 0.90
Modified HBM Scale [27]	Measure HBM constructs	44	44–174	Cronbach’s α = 0.873; test–retest ICC = 0.889
Modified Medical Outcomes Study Social Support Survey (mMOS-SS) [28]	Evaluate instrumental and emotional social support	8	8–40	Cronbach’s α = 0.822; test–retest ICC = 0.88
Financial Hardship Scale (FHS)	Quantify economic toxicity	10	0–10	Cronbach’s α = 0.810; test–retest ICC = 0.87
EQ-5D-5L [29]	Measure health-related quality of life	5 + VAS	0–1	Cronbach’s α = 0.892; test–retest ICC = 0.89
Generalized Anxiety Disorder Scale (GAD-7)	Screen for anxiety symptoms	7	0–21	Cronbach’s α = 0.830; test–retest ICC = 0.86
European Health Literacy Survey Questionnaire-Short Form (HLS-EU-Q16)	Measure general health literacy across healthcare, prevention, and promotion domains	16	16–64	Cronbach’s α = 0.854; test–retest ICC = 0.88; validated in Chinese populations

**Table 2:** Differences in two-dimensional diagnostic adherence by demographic and clinical variables.

Variable	Category	n	Cognitive Dimension (Mean ± SD)	Behavioral Dimension (Completion Rate, %)	Test Statistic (Cog/Beh)	Raw *p*/FDR-Adjusted *p*	Effect Size (Cog/Beh)
Gender	Female	131	5.66 ± 2.66	48.9 (64/131)	t = 5.080/χ^2^ = 5.87	<0.001/<0.001 (cog); 0.015/0.015 (beh)	d = 0.64/OR = 1.83
	Male	117	4.13 ± 2.07	34.2 (41/117)			
Medical Accessibility	≤30 min	126	5.63 ± 2.67	51.6 (65/126)	t = 4.597/χ^2^ = 8.12	<0.001/<0.001 (cog); 0.004/0.004 (beh)	d = 0.59/OR = 2.18
	>30 min	122	4.22 ± 2.12	32.8 (40/122)			
Living Status	With spouse/children	106	5.82 ± 2.48	52.8 (56/106)	F = 42.31/χ^2^ = 15.67	<0.001/<0.001 (cog); <0.001/<0.001 (beh)	η^2^ = 0.26/Cramer’s V = 0.25
	With other family	45	5.18 ± 2.35	46.7 (21/45)			
	Living alone	97	3.78 ± 2.12	28.9 (28/97)			
Comorbidities	Yes	165	5.57 ± 2.29	49.1 (81/165)	t = 5.988/χ^2^ = 9.24	<0.001/<0.001 (cog); 0.002/0.002 (beh)	d = 0.77/OR = 2.36
	No	83	3.67 ± 2.46	29.0 (24/83)			
COPD Family History	Yes	215	5.20 ± 2.44	45.1 (97/215)	t = 4.567/χ^2^ = 5.12	<0.001/<0.001 (cog); 0.024/0.024 (beh)	d = 0.84/OR = 2.59
	No	33	3.15 ± 2.38	24.2 (8/33)			
Smoking Status	Never smoked	112	4.62 ± 2.31	38.4 (43/112)	F = 7.82/χ^2^ = 8.92	<0.001/<0.001 (cog); 0.012/0.012 (beh)	η^2^ = 0.06/Cramer’s V = 0.19
	Former smoker	70	5.78 ± 2.56	54.3 (38/70)			
	Current smoker	66	4.38 ± 2.48	36.4 (24/66)			
Anxiety (GAD-7)	<10	218	5.07 ± 2.52	44.7 (97/218)	t = 2.468/χ^2^ = 4.12	0.014/0.014 (cog); 0.042/0.042 (beh)	d = 0.49/OR = 2.21
	≥10	30	3.87 ± 2.31	26.7 (8/30)			

Note: Cognitive dimension tested with independent samples *t*-test (2 groups) or one-way ANOVA (3+ groups); behavioral dimension tested with chi-square test (or Fisher’s exact test where appropriate). *p*-values are presented as raw/FDR-adjusted. The BH FDR procedure was applied across all 14 group-comparison tests (7 cognitive + 7 behavioral). Effect sizes: Cohen’s d/η^2^ for cognitive; odds ratio/Cramer’s V for behavioral. Total n = 248 for all analyses.

**Table 3:** Pearson correlation matrix of key variables.

Variable	1	2	3	4	5	6	7	8	9
1. Cognitive Dimension	1.00	—	—	—	—	—	—	—	—
2. Behavioral Dimension	0.812 **	1.00	—	—	—	—	—	—	—
3. COPD Knowledge	0.786 **	0.752 **	1.00	—	—	—	—	—	—
4. Health Beliefs	0.788 **	0.736 **	0.764 **	1.00	—	—	—	—	—
5. Social Support	0.723 **	0.701 **	0.689 **	0.712 **	1.00	—	—	—	—
6. Economic Toxicity	−0.630 **	−0.602 **	−0.587 **	−0.614 **	−0.245 **	1.00	—	—	—
7. Health Literacy	0.731 **	0.715 **	0.703 **	0.728 **	0.695 **	−0.598 **	1.00	—	—
8. Anxiety	−0.183 **	−0.157 *	−0.142 *	−0.168 *	−0.135 *	0.142 *	−0.129 *	1.00	—
9. Living Alone	−0.482 **	−0.457 **	−0.423 **	−0.448 **	−0.512 **	0.326 **	−0.417 **	0.189 *	1.00

Note: * *p* < 0.05, ** *p* < 0.001.

**Table 4:** Sociodemographic characteristics of participants.

Characteristic	Category	n (%)	95%CI for Percentage (%)
Gender	Female	131 (52.82)	46.63–59.01
	Male	117 (47.18)	40.99–53.37
Age (years)	40–50	14 (5.65)	2.98–8.32
	51–60	23 (9.27)	5.76–12.78
	61–70	71 (28.63)	22.94–34.32
	≥71	140 (56.45)	49.98–62.92
Education Level	No formal education	37 (14.92)	10.57–19.27
	Primary school	95 (38.31)	32.26–44.36
	Junior high	82 (33.06)	27.15–38.97
	Senior high/technical school	28 (11.29)	7.45–15.13
	College or higher	6 (2.42)	0.46–4.38
Monthly Family Income (RMB)	≤1000	95 (38.31)	32.26–44.36
	1001–2000	102 (41.13)	34.98–47.28
	>2000	51 (20.56)	15.32–25.80
Living Status	Living alone	97 (39.11)	33.06–45.16
	Living with spouse/children	106 (42.74)	36.59–48.89
	Living with other family	45 (18.15)	13.37–22.93
Travel Time to Hospital	<15 min	55 (22.18)	17.01–27.35
	15–30 min	71 (28.63)	22.94–34.32
	>30 min	122 (49.19)	42.86–55.52
Medical Insurance	Yes (Rural Cooperative)	225 (90.73)	87.01–94.45
	No	23 (9.27)	5.55–12.99

**Table 5:** Clinical characteristics and COPD risk factors.

Characteristic	Category	n (%)	95%CI for Percentage (%)
Smoking Status	Never smoker	112 (45.16)	38.91–51.41
	Former smoker	70 (28.23)	22.64–33.82
	Current smoker	66 (26.61)	21.14–32.08
COPD Family History	Yes	215 (86.69)	82.36–91.02
	No	33 (13.31)	8.98–17.64
Biomass Fuel Exposure	Yes (≥4 h/day, ≥5 years)	96 (38.71)	32.66–44.76
	No	152 (61.29)	55.24–67.34
Occupational Exposure	Yes	38 (15.32)	10.98–19.66
	No	210 (84.68)	80.34–89.02
Comorbidities	None	83 (33.47)	27.60–39.34
	Hypertension	132 (53.23)	47.08–59.38
	Type 2 Diabetes	34 (13.71)	9.52–17.90
	Coronary Heart Disease	17 (6.86)	3.83–9.89
	Stroke	10 (4.03)	1.75–6.31
Nighttime Respiratory Symptoms	Yes	144 (58.06)	51.91–64.21
	No	104 (41.94)	35.79–48.09
COPD Health Education	Yes	30 (12.10)	8.06–16.14
	No	218 (87.90)	83.86–91.94

**Table 6:** Reliability and validity indices of study scales.

Scale	Cronbach’s α (Overall)	Test–Retest ICC (95%CI)	KMO	Bartlett’s χ^2^ (df)	*p*
COPD Diagnostic Adherence Literacy Scale	0.828	0.89 (0.81–0.95)	0.798	300.329 (36)	<0.001
COPD-Q	0.710	0.90 (0.83–0.96)	0.723	215.478 (78)	<0.001
Modified HBM Scale	0.873	0.889 (0.80–0.95)	0.921	1892.645 (594)	<0.001
mMOS-SS	0.822	0.88 (0.79–0.94)	0.785	320.517 (28)	<0.001
FHS	0.810	0.87 (0.78–0.93)	0.741	198.763 (45)	<0.001
EQ-5D-5L	0.892	0.89 (0.81–0.95)	0.812	412.678 (90)	<0.001
GAD-7	0.830	0.86 (0.77–0.92)	0.756	245.812 (21)	<0.001

Note: CFA fit indices for the COPD Diagnostic Adherence Literacy Scale (three-factor model, n = 124 independent CFA sample): χ^2^/df = 1.87, CFI = 0.958, TLI = 0.943, RMSEA = 0.059 (90%CI: 0.041–0.078), SRMR = 0.048. Factor loadings ranged from 0.58 to 0.83. EFA was conducted in an independent split-half sample (n = 124). Two-factor vs. one-factor CFA for adherence constructs: two-factor model CFI = 0.942, RMSEA = 0.074, SRMR = 0.052 vs. one-factor CFI = 0.856, RMSEA = 0.112, SRMR = 0.089; Δχ^2^ = 156.3, Δdf = 1, *p* < 0.001.

**Table 7:** Descriptive statistics of key variables.

Variable	Mean ± SD	Range	Median	Q1–Q3	95%CI for Mean
Cognitive Adherence (Literacy Scale)	4.935 ± 2.512	0–9	5.00	3.00–7.00	4.612–5.258
Behavioral Adherence (Coverage Rate, %)	42.3	0–100	—	—	36.1–48.5
COPD-Q (Knowledge)	7.181 ± 3.367	0–13	7.00	5.00–9.00	6.785–7.577
Modified HBM Scale (Total)	107.157 ± 31.803	44–174	105.00	82.00–131.00	103.215–111.099
Perceived Susceptibility	18.543 ± 7.012	6–30	18.00	13.00–24.00	17.721–19.365
Perceived Severity	20.915 ± 7.725	7–35	21.00	15.00–27.00	20.012–21.818
Perceived Benefits	19.876 ± 6.982	6–32	20.00	14.00–25.00	19.053–20.699
Perceived Barriers	18.177 ± 6.714	5–30	18.00	12.00–24.00	17.385–18.969
Self-Efficacy	19.234 ± 7.153	6–33	19.00	13.00–25.00	18.387–20.081
Cues to Action	18.765 ± 6.891	5–31	18.00	13.00–24.00	17.952–19.578
mMOS-SS (Social Support, Total)	24.387 ± 8.872	8–40	24.00	17.00–31.00	23.345–25.429
Instrumental Support	12.044 ± 4.991	4–20	12.00	8–16	11.412–12.676
Emotional Support	12.343 ± 5.045	4–20	12.00	8–16	11.701–12.985
FHS (Economic Toxicity)	4.948 ± 2.644	0–10	5.00	3–7	4.632–5.264
GAD-7 (Anxiety)	4.21 ± 2.89	0–21	4.00	2–6	3.872–4.548
EQ-5D-5L (HRQOL)	0.78 ± 0.15	0.32–1.00	0.80	0.68–0.90	0.761–0.799
Health Literacy (HLS-EU-Q16)	32.47 ± 8.62	16–58	32.00	26.00–39.00	31.39–33.55

**Table 8:** Multiple regression models for diagnostic adherence (linear for cognitive outcome; logistic for behavioral outcome).

Outcome: Cognitive Adherence (Linear Regression)	Predictor	B (SE)	β	t	*p*	95%CI for B
	Health Literacy	0.121 (0.023)	0.286	5.261	<0.001	0.084–0.158
	Health Beliefs	0.020 (0.004)	0.252	4.892	<0.001	0.010–0.030
	COPD Knowledge	0.178 (0.035)	0.239	5.085	<0.001	0.094–0.262
	Living Alone	−0.430 (0.112)	−0.157	−3.839	<0.001	−0.676–−0.184
	Economic Toxicity	−0.103 (0.038)	−0.108	−2.710	0.007	−0.185–−0.021
	Comorbidities	0.362 (0.168)	0.068	2.155	0.032	0.011–0.713
	COPD Family History	0.425 (0.208)	0.065	2.043	0.042	0.003–0.847
	Predictor	B (SE) [log-odds]	OR (95%CI)	Wald χ^2^	*p*	Interpretation
	Health Literacy	0.247 (0.052)	1.28 (1.15–1.42)	22.56	<0.001	Per 1-point increase in HLS-EU-Q16
	Social Support	0.113 (0.040)	1.12 (1.04–1.21)	8.01	0.005	Per 1-point increase in mMOS-SS
	Economic Toxicity	−0.198 (0.069)	0.82 (0.72–0.94)	8.26	0.004	Per 1-point increase in FHS
	Medical Accessibility	0.615 (0.257)	1.85 (1.12–3.05)	5.73	0.017	≤30 min vs. >30 min from hospital

Cognitive model: R^2^ = 0.634, Adjusted R^2^ = 0.623, F = 59.355 (df = 7240), *p* < 0.001; VIF range 1.21–2.43 (mean 1.72); Durbin–Watson = 1.98. Behavioral model: Nagelkerke R^2^ = 0.68, Hosmer–Lemeshow χ^2^ = 7.23 (df = 8, *p* = 0.512), AUC = 0.89 (95%CI: 0.85–0.93); VIF range 1.15–1.89. Regression *p*-values are FDR-adjusted across the predictor family within each model (7 predictors for cognitive, 4 for behavioral); raw *p*-values were identical to adjusted values for all predictors except comorbidities (raw *p* = 0.032, FDR = 0.037) and COPD family history (raw *p* = 0.042, FDR = 0.047) in the cognitive model. B = unstandardized coefficient; β = standardized coefficient; OR = odds ratio; CI = confidence interval.

**Table 9:** Bootstrap-mediated effect tests.

Mediating Path	Dependent Variable	Total Effect (c)	Direct Effect (c’)	Indirect Effect (ab)	SE	95% Bootstrap CI	*p* (FDR)	% of Total Association
Knowledge → Health Beliefs → Adherence	Cognitive Dimension	0.484 **	0.274 **	0.152	0.056	[0.098, 0.317]	<0.001 **	31.4%
	Behavioral Dimension	9.21 **	6.57 **	2.64	0.78	[1.21, 4.18]	<0.001 **	28.7%
Knowledge → Social Support → Adherence	Cognitive Dimension	0.484 **	0.274 **	0.058	0.043	[−0.007, 0.162]	0.068 (ns)	12.0%
	Behavioral Dimension	9.21 **	7.89 **	1.32	0.56	[0.15, 2.49]	0.027 *	18.5%
Economic Toxicity → Health Beliefs → Adherence	Cognitive Dimension	−0.203 **	−0.098 *	−0.075	0.028	[−0.139, −0.030]	0.002 **	36.9%
	Behavioral Dimension	−4.54 **	−2.67 *	−1.87	0.69	[−3.24, −0.50]	0.008 **	41.2%
Economic Toxicity → Social Support → Adherence	Cognitive Dimension	−0.203 **	−0.098 *	−0.030	0.021	[−0.078, 0.002]	0.062 (ns)	14.8%
	Behavioral Dimension	−4.54 **	−3.62 *	−0.92	0.48	[−1.91, 0.07]	0.065 (ns)	20.3%

Note: * *p* < 0.05, ** *p* < 0.001. Covariates include age, education level, and GAD-7 score. Cognitive outcome mediation uses OLS regression for all paths. Behavioral outcome mediation uses logistic regression for the outcome path; indirect effects are estimated on the log-odds scale. For the behavioral outcome, the percentage of total association is computed as ab/c on the log-odds metric, owing to the non-collapsibility of odds ratios; these percentages are not interpretable on the probability scale. E-values follow VanderWeele & Ding (2017) and VanderWeele (2015) [39,40]. All *p*-values are FDR-corrected. ‘ns’ = not statistically significant after FDR correction. E-values for significant indirect pathways range from 1.82 to 2.35. Given the cross-sectional design, all indirect associations are exploratory and should not be interpreted as causal effects.

## Data Availability

The data that support the findings of this study are available from the corresponding authors upon reasonable request. Raw data containing sensitive personal information are protected by ethical regulations and cannot be made publicly available. De-identified data can be accessed by submitting a formal request, including a research proposal, institutional ethics approval, and a data use agreement, to the corresponding authors.

## Supplementary Materials

The following supporting information can be downloaded at: https://www.mdpi.com/article/10.3390/healthcare14193251/s1. Table S1: Full Sociodemographic Characteristics of Participants (N = 248); Table S2: Full Clinical Characteristics and COPD Risk Factors N = 248); Table S3: Full Item Content of the COPD Diagnostic Adherence Literacy Scale with Factor Loadings; Table S4: Detailed EFA and CFA Results for the COPD Diagnostic Adherence Literacy Scale; Figure S1: Reliability Coefficients of Psychometric Scales; Figure S2: Conceptual Mediation Model. The study’s measurement scales and statistical analysis code (for SPSS 27.0, Python 3.9, and R 4.3.1) are also available in the Supplementary Materials.

## Abbreviations:

The following abbreviations are used in this manuscript:

Abbreviation	Full Term
COPD	Chronic Obstructive Pulmonary Disease
HBM	Health Belief Model
LMICs	Low- and Middle-Income Countries
DALYs	Disability-Adjusted Life Years
WHO	World Health Organization
VHW	Village Health Worker
FHS	Financial Hardship Scale
mMOS-SS	modified Medical Outcomes Study Social Support Survey
COPD-Q	COPD Knowledge Questionnaire
GAD-7	Generalized Anxiety Disorder Scale
HRQOL	Health-Related Quality of Life
EQ-5D-5L	EuroQol 5-Dimension 5-Level
EFA	Exploratory Factor Analysis
CFA	Confirmatory Factor Analysis
CVI	Content Validity Index
CVR	Content Validity Ratio
ICC	Intraclass Correlation Coefficient
VIF	Variance Inflation Factor
CI	Confidence Interval
SD	Standard Deviation
RMB	Renminbi
STROBE	Strengthening the Reporting of Observational Studies in Epidemiology
