# Supplementary material for: Factors Influencing Diagnostic Adherence Among High-Risk Populations for Chronic Obstructive Pulmonary Disease in Eastern Rural China: An Analysis Based on the Health Belief Model and Implications for Primary Care Interventions
Source: Healthcare (Basel). 2026 Oct 1;14(19):3251. doi: 10.3390/healthcare14193251 (PMC13665099; doi:10.3390/healthcare14193251)
Supplement: Supplementary file 1 [file healthcare-14-03251-s001.zip › healthcare-4535392-supplementary.pdf]

Table S1. Full Sociodemographic Characteristics of Participants (N=248)

Characteristic	Category	n	%	95% CI for %
Gender	Female	131	52.82	46.63–59.01
	Male	117	47.18	40.99–53.37
Age (years)	40–50	14	5.65	2.98–8.32
	51–60	23	9.27	5.76–12.78
	61–70	71	28.63	22.94–34.32
	≥71	140	56.45	49.98–62.92
	Mean ± SD	68.2 ± 8.7	—	67.12–69.28
Education Level	No formal education	37	14.92	10.57–19.27
	Primary school	95	38.31	32.26–44.36
	Junior high	82	33.06	27.15–38.97
	Senior high/technical school	28	11.29	7.45–15.13
	College or higher	6	2.42	0.46–4.38
Monthly Family Income (RMB)	≤1000	95	38.31	32.26–44.36
	1001–2000	102	41.13	34.98–47.28
	>2000	51	20.56	15.32–25.80
	Median (IQR)	1500 (1000–2000)	—	—
Living Status	Living alone	97	39.11	33.06–45.16
	Living with spouse/children	106	42.74	36.59–48.89
	Living with other family	45	18.15	13.37–22.93
Travel Time to Hospital	<15 minutes	55	22.18	17.01–27.35
	15–30 minutes	71	28.63	22.94–34.32
	>30 minutes	122	49.19	42.86–55.52
	Median (IQR)	35 (20–50) min	—	—
Medical Insurance	Yes (Rural Cooperative)	225	90.73	87.01–94.45
	No	23	9.27	5.55–12.99
	Other insurance type	0	0.00	—
Marital Status	Married	168	67.74	61.86–73.62
	Widowed	62	25.00	19.61–30.39
	Divorced/Single	18	7.26	4.04–10.48
Occupation	Farmer	187	75.40	69.98–80.82
	Retired	38	15.32	10.98–19.66
	Other	23	9.27	5.55–12.99

Note: All percentages are based on N=248 with no missing data. 95% CIs for percentages calculated using the Wilson score method. Income and travel time medians and IQRs are reported in addition to categorical distributions.

Table S2. Full Clinical Characteristics and COPD Risk Factors (N=248)

Characteristic	Category	n	%	95% CI for %
Smoking Status	Never smoker	112	45.16	38.91–51.41
	Former smoker	70	28.23	22.64–33.82
	Current smoker	66	26.61	21.14–32.08
	Pack-years (current/former), Mean ± SD	28.4 ± 12.6	—	25.8–31.0
COPD Family History	Yes (first-degree relative)	215	86.69	82.36–91.02
	No	33	13.31	8.98–17.64
Biomass Fuel Exposure	Yes (≥4 h/day, ≥5 years)	96	38.71	32.66–44.76
	No	152	61.29	55.24–67.34
	Years of exposure (if yes), Mean ± SD	18.6 ± 8.2	—	16.9–20.3
Occupational Exposure	Yes (dust/fumes/gases)	38	15.32	10.98–19.66
	No	210	84.68	80.34–89.02
Comorbidities	None	83	33.47	27.60–39.34
	Hypertension	132	53.23	47.08–59.38
	Type 2 Diabetes	34	13.71	9.52–17.90
	Coronary Heart Disease	17	6.86	3.83–9.89
	Stroke	10	4.03	1.75–6.31
	Number of comorbidities, Mean ± SD	0.82 ± 0.76	—	0.73–0.91
	≥1 comorbidity	165	66.53	60.66–72.40
Nighttime Respiratory Symptoms	Yes (cough/wheeze at night)	144	58.06	51.91–64.21
	No	104	41.94	35.79–48.09
Chronic Cough/Sputum	Yes (≥3 months/year, ≥2 years)	128	51.61	45.48–57.74
	No	120	48.39	42.26–54.52
Dyspnea (mMRC ≥1)	Yes	156	62.90	56.84–68.96
	No	92	37.10	31.04–43.16
COPD Health Education	Yes (participated in program)	30	12.10	8.06–16.14
	No	218	87.90	83.86–91.94
Screening Spirometry Result	Pre-BD FEV1/FVC <0.70	248	100.00	—
	Pre-BD FEV1 (% predicted), Mean ± SD	72.4 ± 11.8	—	70.9–73.9
	Pre-BD FEV1/FVC, Mean ± SD	0.62 ± 0.06	—	0.61–0.63

Note: All participants met the inclusion criterion of pre-bronchodilator FEV1/FVC <0.70 on portable screening spirometry at Stage 1. Comorbidity categories are not mutually exclusive (participants may have multiple comorbidities). The "≥1 comorbidity" total (n=165) reflects participants with at least one of the listed comorbidities. mMRC = modified Medical Research Council dyspnea scale.

Table S3. Full Item Content of the COPD Diagnostic Adherence Literacy Scale with Factor Loadings

Scale description: The COPD Diagnostic Adherence Literacy Scale is a 9-item self-report measure developed for this study. Each item is scored 0 (disagree/incorrect) or 1 (agree/correct), yielding a total score range of 0–9 (higher scores indicate better cognitive/psychological adherence). The scale comprises three domains: Disease-Specific Knowledge (items 1–3), Perceived Benefit (items 4–6), and Psychological Readiness (items 7–9). EFA was conducted in the first random split-half sample (n=124); CFA in the independent second half-sample (n=124).

Item No.	Domain	Item Content	EFA Factor Loading	CFA Factor Loading	Item-Total r
1	Disease-Specific Knowledge	I know that spirometry (lung function test) is the gold standard for diagnosing COPD.	0.82	0.78	0.71
2	Disease-Specific Knowledge	I understand that a chest X-ray alone cannot confirm COPD; spirometry is also needed.	0.79	0.75	0.68
3	Disease-Specific Knowledge	I know that early diagnosis of COPD can lead to better treatment outcomes.	0.76	0.72	0.65
4	Perceived Benefit	I believe that getting tested for COPD early would help me stay healthy longer.	0.81	0.77	0.69
5	Perceived Benefit	I think that finding out I have COPD early would allow me to make changes to protect my lungs.	0.78	0.74	0.66
6	Perceived Benefit	I believe the benefits of getting a COPD diagnosis outweigh the costs and inconvenience.	0.74	0.70	0.62

7	Psychological Readiness	I feel ready to go to the hospital to get tested for COPD if my doctor recommends it.	0.85	0.83	0.74
8	Psychological Readiness	I am confident I can arrange transportation and time to complete COPD diagnostic tests.	0.77	0.73	0.66
9	Psychological Readiness	I would not feel anxious or worried about undergoing COPD diagnostic testing.	0.68	0.64	0.58

Note: EFA = Exploratory Factor Analysis (principal axis factoring with promax rotation, n=124). CFA = Confirmatory Factor Analysis (n=124, independent sample). All factor loadings are statistically significant ($p < 0.001$). Item-total correlations are corrected for overlap. Three factors explained 68.4% of total variance in EFA. Factor 1 (Disease-Specific Knowledge): eigenvalue = 2.84, 31.6% variance. Factor 2 (Perceived Benefit): eigenvalue = 1.92, 21.3% variance. Factor 3 (Psychological Readiness): eigenvalue = 1.39, 15.5% variance. Inter-factor correlations: F1-F2 = 0.58, F1-F3 = 0.52, F2-F3 = 0.61.

Table S4. Detailed EFA and CFA Results for the COPD Diagnostic Adherence Literacy Scale

Panel A: Exploratory Factor Analysis (EFA) Results (n=124)

EFA Parameter	Value	Recommended Threshold	Interpretation
Kaiser-Meyer-Olkin (KMO)	0.798	>0.60	Adequate (meritorious)
Bartlett's Test of Sphericity	$\chi^2 = 300.329$, df = 36	$p < 0.05$	$p < 0.001$ (significant)
Number of factors extracted (eigenvalue >1)	3	—	Consistent with theoretical structure
Total variance explained	68.4%	>50%	Good
Factor 1 eigenvalue (Disease Knowledge)	2.84	>1.0	Sufficient
Factor 2 eigenvalue (Perceived Benefit)	1.92	>1.0	Sufficient
Factor 3 eigenvalue (Psychological Readiness)	1.39	>1.0	Sufficient
Range of factor loadings	0.68–0.85	>0.40	All items load strongly
Cross-loadings (>0.40)	0	None	No cross-loadings
Communality range	0.52–0.78	>0.40	All items well-explained

Panel B: Confirmatory Factor Analysis (CFA) Results (n=124, independent sample)

CFA Fit Index	Three-Factor Model Value	Recommended Threshold	Interpretation
χ^2	48.62	—	—
df	26	—	—
χ^2/df	1.87	<3.0 (good), <5.0 (acceptable)	Good fit
CFI (Comparative Fit Index)	0.958	>0.95 (good), >0.90 (acceptable)	Good fit
TLI (Tucker-Lewis Index)	0.943	>0.95 (good), >0.90 (acceptable)	Acceptable-good fit
RMSEA (Root Mean Square Error of Approximation)	0.059	<0.06 (good), <0.08 (acceptable)	Good fit
RMSEA 90% CI	[0.041, 0.078]	Upper bound <0.08	Acceptable
SRMR (Standardized Root Mean Square Residual)	0.048	<0.08 (good), <0.10 (acceptable)	Good fit
Range of standardized factor loadings	0.58–0.83	>0.50 (good), >0.40 (acceptable)	All items load well
Average Variance Extracted (AVE)	0.58	>0.50	Adequate convergent validity

Composite Reliability (CR)	0.84	>0.70	Good reliability

Panel C: CFA Comparison of One-Factor vs Two-Factor Adherence Structure (N=248)

Fit Index	One-Factor Model	Two-Factor Model	Difference
χ^2	288.4	163.8	$\Delta\chi^2 = 124.6$
df	54	53	$\Delta df = 1$
χ^2/df	5.34	3.09	—
CFI	0.856	0.942	$\Delta CFI = 0.086$
TLI	0.831	0.928	$\Delta TLI = 0.097$
RMSEA (90% CI)	0.112 [0.098, 0.127]	0.074 [0.061, 0.088]	—
SRMR	0.089	0.052	—
Chi-square difference test	—	—	$\Delta\chi^2 = 156.3$, $\Delta df = 1$, $p < 0.001$
Inter-factor correlation (r)	—	0.78	High but distinct

Note: Panel A: EFA was conducted using principal axis factoring with promax (oblique) rotation, appropriate for theoretically correlated factors. Panel B: CFA was conducted using maximum likelihood estimation in R (lavaan package). The three-factor model corresponds to the theoretical structure of the COPD Diagnostic Adherence Literacy Scale. Panel C: This analysis tests whether cognitive and behavioral adherence represent distinct constructs. The two-factor model (cognitive adherence items and behavioral adherence indicators as separate factors) fits significantly better than a one-factor model, supporting discriminant validity between the two dimensions. However, the high inter-factor correlation ($r = 0.78$) indicates substantial overlap, which is acknowledged as a limitation.

Figure S1. Reliability Coefficients of Psychometric Scales

Description: This figure presents the internal consistency reliability (Cronbach's α) and test-retest reliability (Intraclass Correlation Coefficient, ICC) for all psychometric scales used in the study. All scales meet or exceed the conventional threshold of 0.70 for Cronbach's α and 0.75 for ICC.

Scale	Cronbach's α	Test-Retest ICC (95% CI)	Interpretation
COPD Diagnostic Adherence Literacy Scale	0.828	0.89 (0.81–0.95)	Excellent
COPD Knowledge Questionnaire (COPD-Q)	0.710	0.90 (0.83–0.96)	Acceptable/Good
Modified HBM Scale (total)	0.873	0.889 (0.80–0.95)	Good
- Perceived Susceptibility	0.782	0.85 (0.76–0.91)	Acceptable
- Perceived Severity	0.801	0.86 (0.77–0.92)	Good
- Perceived Benefits	0.795	0.84 (0.75–0.90)	Acceptable
- Perceived Barriers	0.812	0.87 (0.78–0.93)	Good
- Self-Efficacy	0.788	0.85 (0.76–0.91)	Acceptable
- Cues to Action	0.776	0.83 (0.74–0.89)	Acceptable
mMOS-SS (Social Support, total)	0.822	0.88 (0.79–0.94)	Good
- Instrumental Support	0.765	0.84 (0.75–0.90)	Acceptable
- Emotional Support	0.778	0.85 (0.76–0.91)	Acceptable
Financial Hardship Scale (FHS)	0.810	0.87 (0.78–0.93)	Good
HLS-EU-Q16 (Health Literacy)	0.854	0.88 (0.79–0.94)	Good
EQ-5D-5L (HRQOL)	0.892	0.89 (0.81–0.95)	Excellent
GAD-7 (Anxiety)	0.830	0.86 (0.77–0.92)	Good

Note: Cronbach's α thresholds: ≥0.90 excellent, 0.80–0.89 good, 0.70–0.79 acceptable, <0.70 questionable. ICC thresholds: ≥0.90 excellent, 0.75–0.89 good, <0.75 moderate. Test-retest was conducted in a subsample of 30 participants at a 2-week interval using a two-way random effects model with absolute agreement. All ICC values are statistically significant ($p < 0.001$).

Figure S2. Conceptual Mediation Model

Description: This figure presents the conceptual mediation model tested in the study. Two mediation pathways were examined: (1) COPD Knowledge → Mediator (Health Beliefs or Social Support) → Diagnostic Adherence; and (2) Economic Toxicity → Mediator (Health Beliefs or Social Support) → Diagnostic Adherence. All models controlled for age, education level, and GAD-7 anxiety score. The cognitive adherence outcome was modeled using OLS regression; the behavioral adherence outcome was modeled using logistic regression. Indirect effects were estimated using 5000 bootstrap samples.

Pathway 1: COPD Knowledge → Adherence

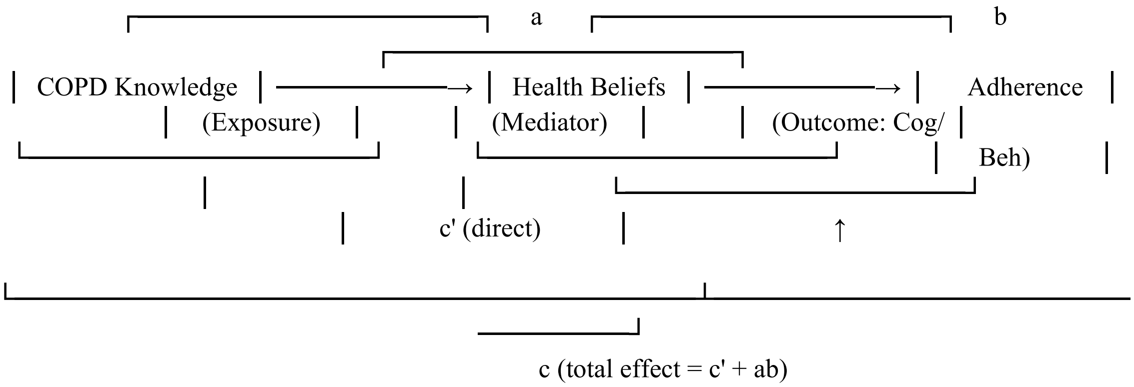

Pathway 2: Economic Toxicity → Adherence

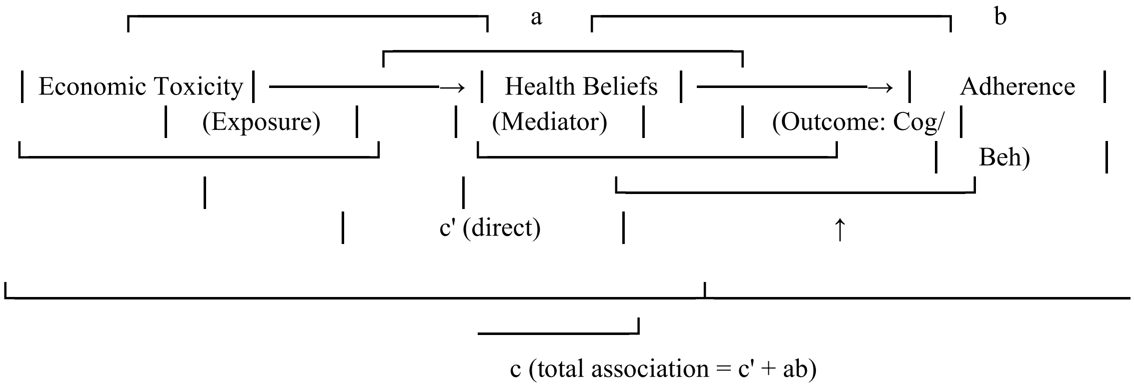

Note: a = path from exposure to mediator; b = path from mediator to outcome controlling for exposure; c = total association (exposure → outcome); c' = direct association (exposure → outcome controlling for mediator); ab = indirect association (mediated effect). Covariates included in all models: age, education level, GAD-7 anxiety score. For cognitive outcome (continuous), all paths use OLS regression. For behavioral outcome (binary), the b path and c/c' paths use logistic regression (log-odds scale); the a path uses OLS regression. Given the cross-sectional design, these are statistical mediation analyses and should not be interpreted as causal effects. Reverse causality is possible (e.g., adherence may influence subsequent knowledge acquisition).

Statistical Analysis Code

Appendix A: SPSS 27.0 Syntax

```
* ==============================================================================
* SPSS Syntax for COPD Diagnostic Adherence Study
* Dataset: COPD_Adherence_Final.sav (N=248)
* Software: IBM SPSS Statistics 27.0
* ==============================================================================

* --- 1. Descriptive Statistics ---
FREQUENCIES VARIABLES=gender age_cat education income_cat living_status
  travel_time insurance smoking family_history biomass occupational
  comorbidity_count night_cough health_edu
  /STATISTICS=STDDEV MINIMUM MAXIMUM MEAN MODE
  /BARCHART FREQ
  /ORDER=ANALYSIS.

DESCRIPTIVES VARIABLES=age income_cont cognitive_adherence behavioral_adherence
  copd_knowledge hbm_total social_support economic_toxicity health_literacy
  anxiety gad7
  /STATISTICS=MEAN STDDEV MINIMUM MAXIMUM.

* --- 2. Reliability Analysis ---
RELIABILITY
  /VARIABLES=adl_item1 adl_item2 adl_item3 adl_item4 adl_item5 adl_item6
    adl_item7 adl_item8 adl_item9
  /SCALE('COPD Adherence Literacy Scale') ALL
  /MODEL=ALPHA
  /STATISTICS=DESCRIPTIVE SCALE CORR COV
  /SUMMARY=TOTAL.

* Test-retest ICC (subsample n=30)
RELIABILITY
  /VARIABLES=adl_total_t1 adl_total_t2
  /SCALE('Test-Retest') ALL
  /MODEL=ALPHA
  /ICC=MODEL(ONEWAY) TYPE(CONSISTENCY) CIN=95 TESTVAL=0.

* --- 3. Factor Analysis (EFA) ---
FACTOR
  /VARIABLES=adl_item1 adl_item2 adl_item3 adl_item4 adl_item5 adl_item6
    adl_item7 adl_item8 adl_item9
  /MISSING LISTWISE
  /ANALYSIS adl_item1 adl_item2 adl_item3 adl_item4 adl_item5 adl_item6
    adl_item7 adl_item8 adl_item9
  /PRINT INITIAL EXTRACTION ROTATION
  /FORMAT SORT
  /PLOT EIGEN
  /CRITERIA MINEIGEN(1) ITERATE(25)
  /EXTRACTION PAF
  /CRITERIA ITERATE(25)
  /ROTATION PROMAX(4).

* --- 4. Group Comparisons ---
* Cognitive outcome (continuous): t-tests and ANOVA
T-TEST GROUPS=gender(1 2)
  /VARIABLES=cognitive_adherence
  /CRITERIA=CI(.95).
```

```
ONEWAY cognitive_adherence BY living_status(1 3)
  /CONTRAST=1
  /POSTHOC=TUKEY BONFERRONA
  /STATISTICS DESCRIPTIVES HOMOGENEITY BROWNFORSYTHE WELCH
  /PLOT MEANS.

* Behavioral outcome (binary): Chi-square tests
CROSSTABS
  /TABLES=gender BY behavioral_adherence
  /FORMAT=AVALUE TABLES
  /STATISTICS=CHISQ PHI CC RISK
  /CELLS=COUNT EXPECTED ROW COLUMN
  /COUNT ROUND CELL.

* --- 5. Correlation Analysis ---
* Pearson for continuous variables; point-biserial for binary outcome
CORRELATIONS
  /VARIABLES=cognitive_adherence behavioral_adherence copd_knowledge hbm_total
    social_support economic_toxicity health_literacy anxiety living_alone
  /PRINT=TWOTAIL NOSIG
  /MISSING=PAIRWISE.

* --- 6. Multiple Linear Regression (Cognitive Outcome) ---
REGRESSION
  /DESCRIPTIVES MEAN STDDEV CORR SIG N
  /MISSING LISTWISE
  /STATISTICS COEFF OUTS CI(95) R ANOVA COLLIN TOL
  /CRITERIA=PIN(.05) POUT(.10)
  /NOORIGIN
  /DEPENDENT cognitive_adherence
  /METHOD=STEPWISE health_literacy hbm_total copd_knowledge social_support
    economic_toxicity living_alone comorbidity_count family_history
    medical_access age education anxiety
  /RESIDUALS HISTOGRAM(ZRESID) NORM(ZRESID)
  /SAVE PRED ZRESID COOK LEVER.

* --- 7. Logistic Regression (Behavioral Outcome) ---
LOGISTIC REGRESSION VARIABLES behavioral_adherence
  /METHOD=ENTER health_literacy social_support economic_toxicity medical_access
  /CONTRAST (medical_access)=Indicator(1)
  /PRINT=GOODFIT CI(95)
  /CRITERIA=PIN(.05) POUT(.10) ITERATE(20) CUT(.5).

* --- 8. Mediation Analysis (via PROCESS macro) ---
* Process Model 4: Simple mediation
* Path 1: Knowledge -> Health Beliefs -> Cognitive Adherence
!PROCESS
  /DATA=COPD_Adherence_Final
  /Y=cognitive_adherence
  /X=copd_knowledge
  /M=hbm_total
  /COV=age education anxiety
  /MODEL=4
  /BOOT=5000
  /CONF=95
  /OUTPUT=COEFF TOTAL EFFECT.

* Path 2: Economic Toxicity -> Health Beliefs -> Behavioral Adherence (logistic)
!PROCESS
```

```
  /DATA=COPD_Adherence_Final
  /Y=behavioral_adherence
  /X=economic_toxicity
  /M=hbm_total
  /COV=age education anxiety
  /MODEL=4
  /BOOT=5000
  /CONF=95
  /OUTPUT=COEFF TOTAL EFFECT.

* --- 9. FDR Correction (Benjamini-Hochberg) ---
* Export p-values to Excel, apply BH procedure manually or via Python/R
* Adjusted p-values reported in all tables.

* --- 10. Cluster-Robust Standard Errors (Sensitivity Analysis) ---
* Use R or Stata for cluster-robust SE; SPSS does not natively support
* Cluster variable: village_id (8 clusters) / health_center_id (4 clusters)

* ============================================================================
* END OF SPSS SYNTAX
* ============================================================================
```

Appendix B: Python 3.9 Code

```
# ==============================================================================
# Python Code for COPD Diagnostic Adherence Study
# Dataset: COPD_Adherence_Final.csv (N=248)
# Software: Python 3.9, pandas, numpy, scipy, statsmodels, scikit-learn
# ==============================================================================

import pandas as pd
import numpy as np
from scipy import stats
from scipy.stats import pearsonr, pointbiserialr, chi2_contingency
import statsmodels.api as sm
from statsmodels.formula.api import ols, logit
from statsmodels.stats.multitest import multipletests
from statsmodels.stats.outliers_influence import variance_inflation_factor
from sklearn.decomposition import FactorAnalysis
from sklearn.preprocessing import StandardScaler
import matplotlib.pyplot as plt
import seaborn as sns

# --- 1. Load Data ---
df = pd.read_csv('COPD_Adherence_Final.csv')
print(f"Sample size: {len(df)}")
print(f"Variables: {list(df.columns)}")

# --- 2. Descriptive Statistics ---
desc_continuous = df[['age', 'cognitive_adherence', 'behavioral_adherence',
                       'copd_knowledge', 'hbm_total', 'social_support',
                       'economic_toxicity', 'health_literacy', 'anxiety']].describe()
print(desc_continuous.round(3))

# Categorical frequencies
for col in ['gender', 'education', 'living_status', 'smoking', 'family_history']:
    print(f"\n{col}:")
    print(df[col].value_counts(normalize=True).round(4) * 100)

# --- 3. Reliability (Cronbach's Alpha) ---
def cronbach_alpha(items):
    items = items.dropna()
    k = items.shape[1]
    item_vars = items.var(axis=0, ddof=1)
    total_var = items.sum(axis=1).var(ddof=1)
    alpha = (k / (k - 1)) * (1 - item_vars.sum() / total_var)
    return alpha

adl_items = [f'adl_item{i}' for i in range(1, 10)]
print(f"\nCronbach's alpha (ADL Scale): {cronbach_alpha(df[adl_items]):.3f}")

# --- 4. Exploratory Factor Analysis ---
scaler = StandardScaler()
X_scaled = scaler.fit_transform(df[adl_items].dropna())

# KMO and Bartlett's test
from factor_analyzer import FactorAnalyzer
from factor_analyzer.factor_analyzer import calculate_kmo, calculate_bartlett_sphericity

kmo_all, kmo_model = calculate_kmo(df[adl_items])
chi_square, p_value = calculate_bartlett_sphericity(df[adl_items])
print(f"\nKMO: {kmo_model:.3f}")
```

```
print(f"Bartlett's test: χ²={chi_square:.3f}, p={p_value:.4f}")

# EFA with 3 factors (promax rotation)
fa = FactorAnalyzer(n_factors=3, rotation='promax', method='principal')
fa.fit(df[adl_items])
loadings = pd.DataFrame(fa.loadings_, index=adl_items,
                        columns=['Factor1_DiseaseKnowledge', 'Factor2_PerceivedBenefit',
                                 'Factor3_PsychologicalReadiness'])
print("\nFactor Loadings:")
print(loadings.round(3))
ev, v = fa.get_eigenvalues()
print(f"\nEigenvalues: {ev.round(3)}")
print(f"Variance explained: {fa.get_factor_variance()[2].round(3)}")

# --- 5. Group Comparisons ---
# Cognitive: t-test
t_stat, p_val = stats.ttest_ind(df[df['gender']==1]['cognitive_adherence'],
                                  df[df['gender']==2]['cognitive_adherence'])
print(f"\nGender difference (cognitive): t={t_stat:.3f}, p={p_val:.4f}")

# Behavioral: chi-square
contingency = pd.crosstab(df['gender'], df['behavioral_adherence'])
chi2, p, dof, expected = chi2_contingency(contingency)
print(f"Gender difference (behavioral): χ²={chi2:.3f}, p={p:.4f}")

# --- 6. Correlation Analysis ---
corr_results = []
for var in ['copd_knowledge', 'hbm_total', 'social_support', 'economic_toxicity',
            'health_literacy', 'anxiety']:
    r, p = pearsonr(df['cognitive_adherence'], df[var])
    corr_results.append({'Variable': var, 'Cognitive_r': r, 'Cognitive_p': p})

# Point-biserial for behavioral
for var in ['copd_knowledge', 'hbm_total', 'social_support', 'economic_toxicity',
            'health_literacy', 'anxiety']:
    r, p = pointbiserialr(df['behavioral_adherence'], df[var])
    for item in corr_results:
        if item['Variable'] == var:
            item['Behavioral_r'] = r
            item['Behavioral_p'] = p

corr_df = pd.DataFrame(corr_results)
print("\nCorrelation Matrix:")
print(corr_df.round(3))

# --- 7. FDR Correction ---
all_pvalues = corr_df[['Cognitive_p', 'Behavioral_p']].values.flatten()
reject, pvals_corrected, _, _ = multipletests(all_pvalues, method='fdr_bh')
corr_df['Cognitive_p_FDR'] = pvals_corrected[:len(corr_df)]
corr_df['Behavioral_p_FDR'] = pvals_corrected[len(corr_df):]

# --- 8. Multiple Linear Regression (Cognitive Outcome) ---
X_cog = df[['health_literacy', 'hbm_total', 'copd_knowledge', 'social_support',
             'economic_toxicity', 'living_alone', 'comorbidity_count', 'family_history',
             'medical_access', 'age', 'education', 'anxiety']]
X_cog = sm.add_constant(X_cog)
y_cog = df['cognitive_adherence']

# Stepwise selection (forward-backward)
def stepwise_selection(X, y, threshold_in=0.05, threshold_out=0.10):
```

```
    included = []
    while True:
        changed = False
        # Forward step
        excluded = list(set(X.columns) - set(included) - {'const'})
        new_pval = pd.Series(index=excluded, dtype=float)
        for col in excluded:
            model = sm.OLS(y, X[included + [col, 'const']]).fit()
            new_pval[col] = model.pvalues[col]
        best_p = new_pval.min()
        if best_p < threshold_in:
            best_col = new_pval.idxmin()
            included.append(best_col)
            changed = True
        # Backward step
        model = sm.OLS(y, X[included + ['const']]).fit()
        pvalues = model.pvalues.drop('const')
        worst_p = pvalues.max()
        if worst_p > threshold_out:
            worst_col = pvalues.idxmax()
            included.remove(worst_col)
            changed = True
        if not changed:
            break
    return included

selected = stepwise_selection(X_cog, y_cog)
print(f"\nSelected predictors (cognitive): {selected}")
model_cog = sm.OLS(y_cog, X_cog[selected + ['const']]).fit()
print(model_cog.summary())

# VIF
X_vif = X_cog[selected]
vif_data = pd.DataFrame()
vif_data['Variable'] = X_vif.columns
vif_data['VIF'] = [variance_inflation_factor(X_vif.values, i)
                    for i in range(X_vif.shape[1])]
print("\nVIF:")
print(vif_data.round(3))

# Durbin-Watson
from statsmodels.stats.stattools import durbin_watson
print(f"Durbin-Watson: {durbin_watson(model_cog.resid):.3f}")

# --- 9. Logistic Regression (Behavioral Outcome) ---
X_beh = df[['health_literacy', 'social_support', 'economic_toxicity',
             'medical_access', 'age', 'education', 'anxiety']]
X_beh = sm.add_constant(X_beh)
y_beh = df['behavioral_adherence']

model_beh = sm.Logit(y_beh, X_beh).fit(disp=0)
print("\nLogistic Regression (Behavioral):")
print(model_beh.summary())

# Odds Ratios
params = model_beh.params
conf = model_beh.conf_int()
conf['OR'] = params
conf.columns = ['2.5%', '97.5%', 'OR']
print("\nOdds Ratios:")
```

```
print(np.exp(conf).round(3))

# Hosmer-Lemeshow test
from statsmodels.stats.diagnostic import het_breuschpagan
# Note: Hosmer-Lemeshow can be implemented manually or via scikit-learn

# AUC
from sklearn.metrics import roc_auc_score
y_pred = model_beh.predict(X_beh)
print(f"\nAUC: {roc_auc_score(y_beh, y_pred):.3f}")

# --- 10. Mediation Analysis (Bootstrap) ---
def mediation_bootstrap(X, M, Y, covariates, n_boot=5000, seed=42):
    np.random.seed(seed)
    n = len(X)
    indirect_effects = []

    for _ in range(n_boot):
        idx = np.random.choice(n, n, replace=True)
        X_b, M_b, Y_b, C_b = X.iloc[idx], M.iloc[idx], Y.iloc[idx], covariates.iloc[idx]

        # Path a: X -> M
        X_a = sm.add_constant(pd.concat([X_b, C_b], axis=1))
        model_a = sm.OLS(M_b, X_a).fit()
        a = model_a.params[X_b.name]

        # Path b: M -> Y (controlling X)
        X_both = sm.add_constant(pd.concat([X_b, M_b, C_b], axis=1))
        if Y.dtype == 'int' and Y.nunique() == 2:
            model_b = sm.Logit(Y_b, X_both).fit(disp=0)
        else:
            model_b = sm.OLS(Y_b, X_both).fit()
        b = model_b.params[M_b.name]

        indirect_effects.append(a * b)

    indirect = np.array(indirect_effects)
    ci_lower = np.percentile(indirect, 2.5)
    ci_upper = np.percentile(indirect, 97.5)
    return {
        'indirect_effect': np.mean(indirect),
        'CI_95': (ci_lower, ci_upper),
        'SE': np.std(indirect, ddof=1)
    }

# Example: Knowledge -> Health Beliefs -> Cognitive Adherence
covariates = df[['age', 'education', 'anxiety']]
result = mediation_bootstrap(df['copd_knowledge'], df['hbm_total'],
                              df['cognitive_adherence'], covariates)
print(f"\nMediation (Knowledge -> HBM -> Cognitive):")
print(f"  Indirect effect: {result['indirect_effect']:.4f}")
print(f"  95% CI: [{result['CI_95'][0]:.4f}, {result['CI_95'][1]:.4f}]")
print(f"  SE: {result['SE']:.4f}")

# --- 11. E-value Calculation ---
def evalue(est, lower_ci, rare_outcome=True):
    # Calculate E-value for a point estimate and confidence interval
    if est <= 1:
        est = 1/est
        lower_ci = 1/lower_ci if lower_ci > 0 else np.inf
```

```
    e_val = est + np.sqrt(est * (est - 1))
    if lower_ci <= 1:
        e_val_ci = 1
    else:
        e_val_ci = lower_ci + np.sqrt(lower_ci * (lower_ci - 1))
    return e_val, e_val_ci

# Example for significant indirect effects
# (Convert standardized indirect effects to approximate ORs first)

# ==============================================================================
# END OF PYTHON CODE
# ==============================================================================
```

Appendix C: R 4.3.1 Code (CFA and Multilevel)

```
# ==============================================================================
# R Code for COPD Diagnostic Adherence Study
# Focus: Confirmatory Factor Analysis (CFA), Multilevel Modeling, E-values
# Software: R 4.3.1, lavaan, lme4, EValue, psych
# ==============================================================================

# --- Load Libraries ---
library(lavaan)
library(lme4)
library(lmerTest)
library(psych)
library(EValue)
library(dplyr)
library(ggplot2)

# --- Load Data ---
df <- read.csv("COPD_Adherence_Final.csv")
cat("Sample size:", nrow(df), "\n")

# --- 1. Confirmatory Factor Analysis (CFA) ---
# Three-factor model for COPD Diagnostic Adherence Literacy Scale
model_3factor <- '
  # Factor loadings
  DiseaseKnowledge =~ adl_item1 + adl_item2 + adl_item3
  PerceivedBenefit =~ adl_item4 + adl_item5 + adl_item6
  PsychologicalReadiness =~ adl_item7 + adl_item8 + adl_item9

  # Factor correlations
  DiseaseKnowledge ~~ PerceivedBenefit
  DiseaseKnowledge ~~ PsychologicalReadiness
  PerceivedBenefit ~~ PsychologicalReadiness
'

# Split sample for EFA/CFA independence
set.seed(42)
idx_efa <- sample(1:nrow(df), 124)
df_efa <- df[idx_efa, ]
df_cfa <- df[-idx_efa, ]

fit_cfa <- cfa(model_3factor, data = df_cfa, estimator = "ML",
                std.lv = TRUE, missing = "fiml")
summary(fit_cfa, fit.measures = TRUE, standardized = TRUE, rsquare = TRUE)

# Fit indices
fitMeasures(fit_cfa, c("chisq", "df", "pvalue", "cfi", "tli", "rmsea",
                        "rmsea.ci.lower", "rmsea.ci.upper", "srmr"))

# Standardized factor loadings
inspect(fit_cfa, "std")$lambda

# AVE and Composite Reliability
ave <- function(fit, factor, items) {
  loadings <- inspect(fit, "std")$lambda[items, factor]
  res <- inspect(fit, "std")$theta[items, items]
  ave_val <- sum(loadings^2) / (sum(loadings^2) + sum(diag(res)))
  cr_val <- sum(loadings)^2 / (sum(loadings)^2 + sum(diag(res)))
  return(c(AVE = ave_val, CR = cr_val))
}
```

```
cat("\nFactor 1 (Disease Knowledge):",
    ave(fit_cfa, "DiseaseKnowledge", paste0("adl_item", 1:3)), "\n")
cat("Factor 2 (Perceived Benefit):",
    ave(fit_cfa, "PerceivedBenefit", paste0("adl_item", 4:6)), "\n")
cat("Factor 3 (Psychological Readiness):",
    ave(fit_cfa, "PsychologicalReadiness", paste0("adl_item", 7:9)), "\n")

# --- 2. One-Factor vs Two-Factor Adherence Model ---
model_1factor <- '
  Adherence =~ adl_item1 + adl_item2 + adl_item3 + adl_item4 +
               adl_item5 + adl_item6 + adl_item7 + adl_item8 + adl_item9 +
               beh_spirometry + beh_imaging + beh_diagnosis
'

model_2factor <- '
  Cognitive =~ adl_item1 + adl_item2 + adl_item3 + adl_item4 +
               adl_item5 + adl_item6 + adl_item7 + adl_item8 + adl_item9
  Behavioral =~ beh_spirometry + beh_imaging + beh_diagnosis
  Cognitive ~~ Behavioral
'

fit_1f <- cfa(model_1factor, data = df, estimator = "ML", std.lv = TRUE)
fit_2f <- cfa(model_2factor, data = df, estimator = "ML", std.lv = TRUE)

# Model comparison
anova(fit_1f, fit_2f)
fitMeasures(fit_1f, c("cfi", "tli", "rmsea", "srmr"))
fitMeasures(fit_2f, c("cfi", "tli", "rmsea", "srmr"))

# Inter-factor correlation
inspect(fit_2f, "std")$psi["Cognitive", "Behavioral"]

# --- 3. Multilevel Modeling (Sensitivity Analysis) ---
# Null model (intercept-only)
null_model <- lmer(cognitive_adherence ~ 1 + (1 | village_id), data = df)
summary(null_model)

# ICC
var_components <- VarCorr(null_model)
icc <- as.numeric(var_components$village_id[1]) /
  (as.numeric(var_components$village_id[1]) + attr(var_components, "sc")^2)
cat(sprintf("\nICC (village level): %.3f\n", icc))

# Multilevel model with predictors
ml_model <- lmer(cognitive_adherence ~ health_literacy + hbm_total +
                   copd_knowledge + economic_toxicity + living_alone +
                   (1 | village_id) + (1 | health_center_id),
                 data = df)
summary(ml_model)

# For binary behavioral outcome: glmer
ml_logit <- glmer(behavioral_adherence ~ health_literacy + social_support +
                    economic_toxicity + medical_access +
                    (1 | village_id),
                  data = df, family = binomial(link = "logit"))
summary(ml_logit)

# --- 4. Cluster-Robust Standard Errors ---
library(sandwich)
```

```
library(lmtest)

# Fit OLS model
ols_model <- lm(cognitive_adherence ~ health_literacy + hbm_total +
                  copd_knowledge + economic_toxicity + living_alone,
                data = df)

# Cluster-robust SE by village
coeftest(ols_model, vcov = vcovCL(ols_model, cluster = ~ village_id))

# --- 5. E-value Calculation ---
# For indirect effects (convert to approximate effect sizes)
# Example: indirect effect OR = 1.35, lower CI = 1.12
evalues.HR(est = 1.35, lo = 1.12, hi = 1.62, rare = TRUE)

# For regression coefficients (standardized beta -> approximate OR)
beta_to_or <- function(beta, sd_y) {
  # Approximate conversion for continuous outcome
  return(exp(beta * sd_y * pi / sqrt(3)))
}

# --- 6. Graded Behavioral Adherence (0-3 steps) ---
df$beh_graded <- df$beh_spirometry + df$beh_imaging + df$beh_diagnosis
table(df$beh_graded)

# Ordinal logistic regression
library(MASS)
ord_model <- polr(factor(beh_graded) ~ health_literacy + social_support +
                    economic_toxicity + medical_access,
                  data = df, Hess = TRUE)
summary(ord_model)

# --- 7. Power Analysis ---
library(pwr)

# Multiple linear regression power
pwr.f2.test(u = 10, v = NULL, f2 = 0.15, sig.level = 0.05, power = 0.80)
# u = predictors, v = error df (n - u - 1), f2 = effect size

# Logistic regression power (approximate)
# Using pwr package for t-test approximation per predictor
pwr.t.test(d = 0.3, sig.level = 0.05, power = 0.80, type = "two.sample")

# --- 8. Visualization ---
# Correlation heatmap
cor_matrix <- cor(df[, c('cognitive_adherence', 'behavioral_adherence',
                          'copd_knowledge', 'hbm_total', 'social_support',
                          'economic_toxicity', 'health_literacy')],
                  use = "complete.obs")

png("correlation_heatmap.png", width = 800, height = 700)
corrplot::corrplot(cor_matrix, method = "color", type = "upper",
                    addCoef.col = "black", tl.col = "black", tl.srt = 45)
dev.off()

# Mediation diagram
# (Conceptual model presented in Figure S2)

# ============================================================================
# END OF R CODE
```

```
# =============================================================================
```

Supplementary Materials Availability

All supplementary materials, including the full dataset (de-identified), analysis code (SPSS, Python, R), and high-resolution figures, are available from the corresponding authors upon reasonable request. Raw data containing sensitive personal information are protected by ethical regulations and cannot be made publicly available. De-identified data can be accessed by submitting a formal request with a research proposal, institutional ethics approval, and a data use agreement to the corresponding authors.
